# Sphingid caterpillars conspicuous patches do not function as distractive marks or warning against predators

**DOI:** 10.1002/ece3.10334

**Published:** 2023-07-23

**Authors:** Julia Barrone, Mayra C. Vidal, Robert Stevenson

**Affiliations:** ^1^ Department of Biology University of Massachusetts Boston Boston Massachusetts USA

**Keywords:** antipredator, artificial prey, caterpillar, distractive marks, protective coloration, conspicuous patches

## Abstract

To avoid predation by visual predators, caterpillars can be cryptic to decrease detectability or aposematic to warn predators of potential unpalatability. However, for some species, it is not clear if conspicuous patches are selected to avoid predation. For example, Pandora sphinx (*Eumorpha pandorus*, Lepidoptera: Sphingidae) caterpillars are assumed to be palatable and have both cryptic (green, brown) and conspicuous (orange, red) color morphs. Five lateral, off‐white to yellow patches on either side may serve as a warning for predators or to draw attention away from the caterpillar's form to function as distractive marks. We conducted a field study in three temperate fragmented forests in Massachusetts to investigate the potential utility of *E. pandorus* coloration and conspicuous patches. Using four plasticine caterpillar prey model treatments, green and red with and without lateral conspicuous patches, we tested the effects of color, patch patterning, and seasonality on attack rates by a variety of taxa. We found that 43% of the prey models (*n* = 964) had bite marks by an array of predators including arthropods (67.5%), birds (18.2%), rodents (11.5%), and large mammals (2.8%). Arthropods as dominant predators align with conclusions from previous studies of prey models placed near ground level. Attack rates peaked for arthropods in late August and early September but were more constant across trials for vertebrates. Arthropods, a heterogeneous group, as indicated by the variety of bite marks, showed significantly higher attack rates on green colored prey models and a tendency of higher attack on solid (non‐patch patterned) prey models. Vertebrates, more visually oriented predators, had significantly higher attack rates on red colored prey models and patch patterned prey models. Thus, our results did not suggest that conspicuous patch patterning reduced predation and therefore, we did not find support for the distractive mark hypothesis or warning hypothesis. Further, our study shows clear contrasting interpretations by different predators regarding visual defensive strategies.

## Graphical abstract

We used the artificial prey technique to test the distractive marks hypothesis and warning hypothesis using the Pandora sphinx caterpillar as a model. Arthropod predators showed significantly higher attack rates on green colored prey models than red but only a tendency of higher attack on solid (non‐patch patterned) prey models, whereas vertebrates, more visually oriented predators, had significantly higher attack rates on red colored and patch patterned prey models. Thus, our results did not suggest that conspicuous patch patterning reduced predation and therefore, we did not find support for the distractive mark hypothesis or warning hypothesis.

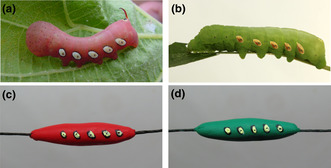

## 1 INTRODUCTION

The glorious array of animal colors and patterns, a prime example of phenotypic variation, has commanded the attention of ecologists and evolutionary biologists for over 100 years (Beddard, 1892; Cott, 1940; Poulton, 1890). In many taxa, color can be used to facilitate anti‐predatory function, such as camouflage and aposematism. Camouflage is a visual mechanism employed by many organisms to remain undetected or unrecognizable if viewed by a predator (Merilaita et al., 2017). Thus, the first step in predation is dependent on detectability of the prey by the predator, or how easily an individual is noticed by a predator (Mand et al., 2007). Visual perception, in the case of both camouflage and warning coloration, depends on both the neural and visual mechanisms of the predator and environmental factors. Considering the latter, Endler (1990, 1993) pointed out that perception depends on ambient light conditions. Tullberg et al. (2005), Barnett et al. (2017), and Barnett et al. (2018) provide examples of distant dependent perception such that when aposematic colors, such as stripes, were viewed within proximity, there was a blatant contrast from the background. However, when viewed from a distance, the aposematic colors increased background blending and concealment. These findings highlight the complexity and context dependence of the success of defensive strategies.

Lepidoptera, both adult and caterpillar forms, have served as model systems to contribute to the importance of predator and environmental conditions regarding color forms and patterning. The ubiquity of Lepidoptera in terrestrial habitats makes them common food items for a variety of natural enemies including birds, mammals, reptiles, and arthropods (Nason et al., 2021). As such, caterpillars have evolved many morphological, chemical, and behavioral defensive strategies to avoid predation such as crypsis, mimicry, masquerading, and aposematism (Ruxton et al., 2004; Skelhorn et al., 2010). For caterpillars, detection by predators may depend on body size (McClure & Despland, 2011), coloration (Skelhorn et al., 2010), pattern (Schuler & Hesse, 1985), and shape (Lichter‐Marck et al., 2015; Suzuki & Sakurai, 2015). For example, Remmel and Tammaru (2009) used artificial prey to conclude that predation risk by avian predators increased with size for both cryptic and conspicuously colored forms. Considering that insects, mainly caterpillars, can constitute a large portion of bird diet (Nyffeler et al., 2018), coloration may be an important trait when avoiding predation by these visual predators. Cryptically colored caterpillars, such as caterpillars that are green or brown, should be less detectable to visual predators due to background matching (Skelhorn et al., 2010). Red and orange colors, on the other hand, are often considered as aposematic warning colors and may increase detectability as they have a greater contrast with the background (Ruxton et al., 2004) and could potentially signal unpalatability to predators. Carroll and Sherratt (2013) found that the aposematically colored moth prey models were more often only partially consumed compared to more cryptically colored prey models, indicating that aposematism may offer a slight defensive advantage over crypsis in some systems.

Additional examples of conspicuous coloration that are commonly found on caterpillars include contrasting markings (Wagner, 2005) and eyespots (Hossie & Sherratt, 2012; Stevens, 2005). There is ample literature on both the evolution and utility of eyespots (Kjernsmo & Merilaita, 2013; Monteiro, 2015; Stevens, 2005; Stevens, Hardman, & Stubbins, 2008) but a lack regarding contrast markings. There are two hypotheses proposed to explain the mechanism by which contrast markings can function as predator avoidance: disruptive coloration and distractive marks (Cott, 1940; Thayer, 1909). Disruptive coloration works to decrease recognition of prey by obstructing the outline (Stevens & Merilaita, 2009), whereas distractive marks work to draw attention to the markings instead of the prey's outline (Stevens, Troscianko, et al., 2013). A few studies have found support for the disruptive color hypothesis (Cuthill et al., 2005; Duarte et al., 2019; Stevens et al., 2006) and distractive marks (Dimitrova et al., 2009; Olofsson et al., 2013) in decreasing predation risk. However, Stevens, Graham, et al. (2008) did not find support for the distractive marks hypotheses and empirical testing for the distractive marks hypothesis is lacking.

The lack of studies and mixed results for distractive marks suggested that studying Pandora sphinx (*Eumorpha pandorus*) caterpillar coloration might provide fruitful insights on predation rate because of its lateral conspicuous patches and cryptic and conspicuous color morphs (Wagner, 2005). Thus, this species combines both cryptic and conspicuous coloration with potential conspicuous patch patterning. Although red and orange hues tend to serve as warning colors in caterpillars, both morphotypes are presumed to be palatable since they lack toxic or chemical defenses. This species utilizes host plants in the Vitaceae family and have a singular posterior eyespot when the anal horn falls off and lateral patches on the thorax and abdomen. The Pandora sphinx caterpillar has five, white to yellow patches encircling the spiracles on either side (Wagner, 2005). The patches differ from eyespots in that they are ovular and less detailed. However, these caterpillars do have a singular posterior eyespot in addition to the lateral patches (Wagner, 2005). Ponce et al. (2015), using 23 *Eumorpha* species, performed a phylogenetic analysis that revealed that an ancestor of *Eumorpha* most likely had a singular posterior eyespot and maintained in most lineages but known to be lost in at least three. Here we report tests for the potential functionality of the patches as an anti‐predatory trait under the distractive marks hypothesis and if color and patch patterning interact to help decrease predation. To single out the visual cue, we used artificial caterpillar prey models. The technique involves deploying artificial prey, made of either clay or dough, (Sam et al., 2015) in a habitat for a designated amount of time (Hitchcock, 2004; Hossie & Sherratt, 2012; Lövei & Ferrante, 2017; Remmel & Tammaru, 2009). When predators attack the artificial prey, they leave imprints. This method provides valuable data to assess predation pressures and targets visual predators because artificial prey cannot replicate behavioral or chemical cues deployed by live caterpillars (Howe et al., 2009).

Our study addresses two central questions: First, do lateral conspicuous patches influence attack rate on caterpillars? Second, do lateral conspicuous patches, on cryptic (green) or conspicuous (red) color morphs have a similar effect on caterpillar attack rate? We aimed to gain insight on the mechanistic function of the patches through attack rates on the different treatments. If there are lower attack rates on green prey models with patches, this would support drawing attention away from the caterpillar's outline under the distractive marks hypothesis. Conversely, if there are lower attack rates on red prey models with patches, this would support avoidance by warning. Additionally, we compared attack rates at different body locations, such that more attacks where the patches are would support the hypothesis of distractive markings. We hypothesized that there would be no difference in attack rate for red over green prey models because of varying detection and attraction factors. Green caterpillars are more cryptic and are often seen as palatable and undefended whereas red prey models may be avoided due to aposematism or could serve as an attractant as various fruits and berries can be red. We supplemented our field experiment with bird surveys, camera traps to survey vertebrate predators, iNaturalist data to gain insight on Pandora sphinx caterpillar color morph frequency, and night surveys to gain a sense of live caterpillar densities at the sites.

## 2 METHODS

### 2.1 Study site selection

We selected three sites in Massachusetts with sufficiently sized Virginia creeper (*Parthenocissus quinquefolia*, Vitaceae) host plant clusters: Breakheart Reservation (42°29′01.4″ N, 71°01′55.6″ W, 652 acres; here after BH) in Saugus, Great Brook Farm State Park (42°33′10.3″ N, 71°20′55.7″ W, 1000 acres; here after GB) in Carlisle, and Bradley Palmer State Park (42°38′58.6″ N, 70°53′55.3″ W, 721 acres; here after BP) in Ipswich (Appendix S1: Figure S1). All sites are temperate forest habitats consisting of both deciduous and coniferous species and used by the public recreationally. A portion of GB is designated as a working dairy farm. Both GB and BP are surrounded by agricultural land and conservation land. BH is bordered by urbanized areas, both housing and commercial. BH and BP have greater amounts of impervious surface trails running through the forest than GB. The sites are a minimum distance of 24 km and a maximum distance of 41 km away from each other. Preliminary surveys showed each site contained at least 16 clusters of Virginia creeper, each cluster measuring a minimum size of 1.25 m × 1.25 m to permit appropriate spacing of at least 1 m between each caterpillar prey model to ensure independence (Roslin et al., 2017). Clusters were separated by a minimum of 10 m, and at least 50 m from the forest edge to avoid edge effects. To record and label the locations of host plants at each site, we used a GPS device (Garmin map 64sx) and dropped pins on Google maps. To ensure randomization, we assigned identified host plant clusters a number from 1 to 16+ at each field site and using a random number generator (https://numbergenerator.org/), selected 14 numbers.

### 2.2 Artificial models

**FIGURE 1:**
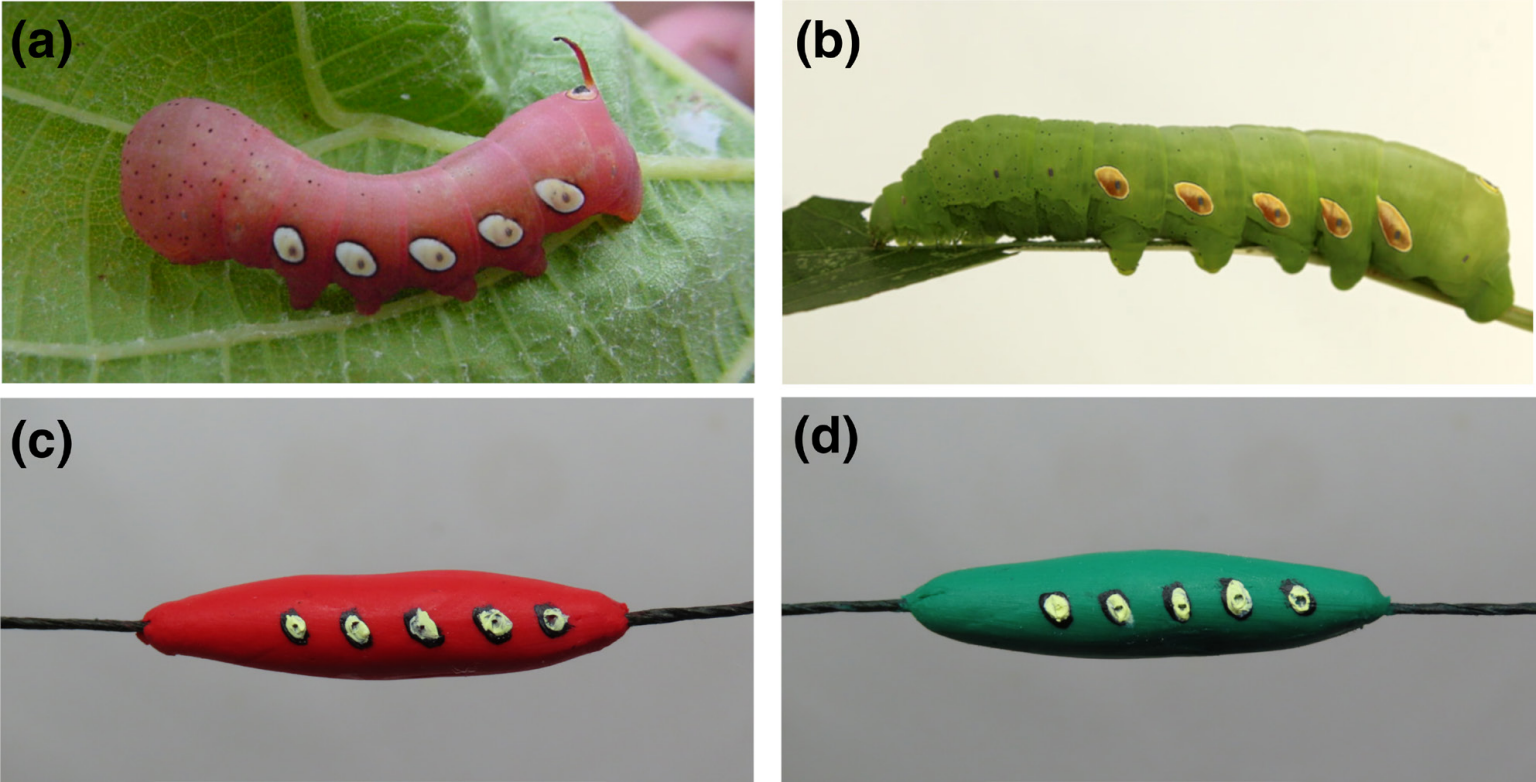
Comparison of live prey and artificial prey. Live (top) and artificial models (bottom) of Pandora sphinx caterpillars. (a) Red color morph (© Duke Elsner) (b) Green color morph (© Teá Montagna) (c) Clay prey model of red color morph. (d) Clay prey model of green color morph. Bottom images (© Julia Barrone).

Artificial caterpillar prey models were formed from non‐hardening and non‐toxic plasticine modeling clay (Van Aken Plastalina Modeling Clay, Van Aken International, North Charleston, SC). We utilized green (cryptic) and red (conspicuous) to form the models, following potential color variation in Pandora sphinx caterpillars (Figure 1). Each caterpillar prey model measured 35 mm in length and 5–6 mm in width as this size of artificial caterpillar was found to be preyed on by both invertebrates and vertebrates (Lövei & Ferrante, 2017), and is representative of the later instar, L4, of Pandora sphinx.

The lateral conspicuous patch patterning was replicated on the artificial caterpillars using black, white, and yellow non‐toxic oil‐based paint (Ohuhu Oil Paint Set). Each patch measured 2 mm and a toothpick was used to create a small indent within the oval to mimic the spiracle. We threaded each prey model longitudinally with a dark green floral wire, leaving 25 mm of wire at either end of the caterpillar to attach the prey model to the host plant (26‐gauge paper coated floral wire), similar to methods used by Roels et al. (2018). During periods of rain and winds, the wires were successful in keeping the prey models fastened to the host plant, but occasionally the prey models detached from the host plant during attacks by large mammals (.83%, *n* = 8). Gloves were worn at all times when handling the prey models.

To gain a sense of spectral properties of the prey models and how well they matched the background under field conditions, we took images of each prey model treatment at one of the Virginia creeper clusters at BH with a gray standard. The field photos were taken post hoc in early June, 2 years after the study was conducted. We then analyzed the photos in ImageJ (Schneider et al., 2012) using the MICA toolbox (Troscianko & Stevens, 2015; van den Berg et al., 2020) and created a color map based on human vision. Color map results are included in our supplementary material (Appendix S1: Table S1 & Figure S2).

### 2.3 Prey model deployment and recovery

One of each of the four prey model variants (green, red, and each with patches) were placed randomly in a host plant cluster attached to the petioles of Virginia creeper, 5–20 cm above ground, depending on the height and location of the host plant. The petiole is an ecologically relevant placement for the prey models as live caterpillars have been observed feeding and resting at this location on host plants (Heinrich, 1979). Fifty‐six prey models were deployed at each site totaling 168 prey models during each trial. All prey models were deployed on the same day, and on days with no heavy rain. We recorded videos of the exact locations of each prey model in the plots to aid in the recovery of the prey models during collection. Each prey model was only deployed once.

We aimed for 50% of the prey models to be attacked as less would have lowered statistical power and greater than 50% would have eroded the treatment effects (Dr. Thomas Hossie, pers comm.). Based on a pilot study, we determined that a week was sufficient time for about half of the prey models to be attacked. Prey models were collected from the field after 1 week following the same order as deployment. We ran six experimental trials at each site from July 17, 2021 through October 17, 2021 to capture the seasonality of predation activity. At each site, we allowed 1–2 weeks to elapse between each trial to minimize learning by predators that the artificial caterpillars did not offer a food reward. A total of 1008 artificial caterpillars were deployed (four prey models per cluster, 14 clusters per site, three sites, six trials). Of these total 1008 prey models, three went missing and five were presumed to have been stepped on and therefore excluded (recovery rate of 99.7%). An additional 36 prey models (11 showed bite marks) that had acrylic, water‐based paint wash off (the paint was subsequently changed to oil‐based as noted above) during rainy conditions in trial one were excluded, leaving 964 prey models (95.6%) for analysis.

### 2.4 Prey model scoring

Predator categories included birds, mammals, and arthropods. We subdivided mammals into non‐rodent and rodent categories as well since rodents can be distinguished from large mammals by smaller tooth sizes and parallel markings of upper and lower incisors. We were unable to determine if multiple bite marks were made by a single predator or multiple predators of the same species, likely yielding conservative estimates of predation in given habitats (Brodie, 1993; Lövei & Ferrante, 2017). Multiple bite marks of the same type of predator were recorded as a single attack on the prey model, while marks from two different predators on a single prey model were scored as separate predation events. However, when analyzing total predator predation by all taxa, we decided to include only one predator per prey model (see data analysis).

We inspected and then collected each prey model in the field. If field team members agreed there were discernible marks, potential signs of predation, the prey model was photographed. We assigned preliminary identifications of bite marks but in the lab, we further examined and photographed the prey models with a digital microscope (Bysameyee HD 2MP USB Microscope, 40× to 1000×) to identify bite marks that were overlooked during the initial field inspection. We also recorded the location of bite marks (classified as end, middle, or both) on each prey model. The ends included the anterior and posterior regions of the prey model and the middle included any region in between the anterior and posterior regions.

Final predator identity was determined by a consensus of three observers. All observers used the bite mark reference guide published by Low et al. (2014) and completed their evaluations separately. ID discrepancies were discussed as a group and a predator ID was accepted if all observers agreed. Unknowns (*n* = 52) were included in the analysis but considered not predated because we could not determine with confidence if an unknown mark was due to an actual predator or made by an object in the habitat. The statistical analyses (see below) were run with and without unknowns included in the dataset and the main results held.

### 2.5 Weekly attack rates

Because prey model placement and duration in the field varies by study, a standard way to compare across studies is to calculate attack rates. We calculated weekly attack rates for all predator taxa for each site per experimental trial and in total for each trial (total attacked/total number of recovered caterpillars per site or trial).

### 2.6 Pandora sphinx caterpillar color frequencies and occurrence at study sites

Because there is a general lack of knowledge about Sphingid color morphs in the wild (Fink, 1989), we downloaded 4602 observations (research‐graded & needs ID) of Pandora sphinx caterpillars on March 6, 2022, from the iNaturalist website (https://www.inaturalist.org). We removed duplicates, (determined by duplicate photographs or identical date and time), pupae (*n* = 73), 1st and 2nd instar caterpillars, which do not exhibit variation in color form (*n* = 7), and months with small sample sizes (March (*n* = 1), April (*n* = 1), May (*n* = 6), and November (*n* = 7), resulting in 1478 observations of caterpillars 3rd instar or older). We then scored caterpillar colors as green, brown, orange, or red. We used a multinomial logistic regression (multinom function in the nnet package, Venables & Ripley, 2002), to model the probability of color as a function of month with brown as the baseline color and June as the baseline month. We also ran a chi‐squared test if the occurrence of each color morph varies by month.

To search for live *Eumorpha* caterpillars and gain a sense of the densities of caterpillars at the sites, we conducted six nocturnal surveys by examining Virginia creeper and Fox grape (*Vitis labrusca*) host plants with a UV flashlight (McDoer Blacklight Flashlight UV 109 LED, 18 W, 385–395 nm) because caterpillar integument fluoresces under UV lighting and caterpillars often actively feed at night. Search times were between 10 pm and 12 am from late August until late September, in which GB was visited three times, BH and BP were each visited twice. The duration of each survey was about 1.5 h and the distance traveled ranged from 0.8 to 1.6 km. Surveys were conducted on trails and mostly near clusters at each site. A total of eight caterpillars in four families were found (Sphingidae, Noctuidae, Erebidae, and Limacodidae, Appendix S1: Table S2) but no Pandora or Achemon sphinx caterpillars were discovered. These findings translate to an approximate caterpillar density of 0.0015 caterpillar hour^−1^ m^−2^ at BH, 0.00042 caterpillar hour^−1^ m^−2^ at GB, and 0.00021 caterpillar hour^−1^ m^−2^ at BP. However, we were secured in using these sites because *Eumorpha* species have been observed in the vicinity of the sites, based on iNaturalist records.

### 2.7 Bird point counts

We performed point count surveys to identify the communities of insectivorous bird species found in each habitat that could influence attack rates. At each site, we randomly selected a 150 m transect near the Virginia creeper clusters over which to conduct the 10 min point counts. All point counts were a minimum of 50 m away from the forest edge and were conducted at 0, 50, and 150 m along the transect. The primary author conducted bird counts at each site four times at the end of July, mid‐end of August, early September, and early October of 2021 during clear weather days. All bird counts were conducted between sunrise and 9:00 am. Bird surveys were conducted at BH and BP on the same days and within a day or two at GH, due to time constraints. Furthermore, a digital audio recorder (Zoom H1n Portable Recorder) with an external microphone (Edutige ETM‐001) was used to record bird songs during point counts at each site. Bird identification applications, Merlin and BirdNet, were also used. The audio was then reviewed in the lab to confirm identification. We filtered the species list to include insectivorous birds that forage on the ground using Birds of the World (Billerman et al., 2022).

### 2.8 Camera traps

To help identify potential predators attacking the prey models, we placed three wildlife camera traps (Campark Upgrade T70 Trail Camera) at BP and two at GB, 5–20 cm above ground, with the view frame focused on one caterpillar prey model during each trial. We did not place camera traps at BH due to reported high rates of vandalism. Each camera had infrared capabilities to record nocturnal predator activity. We reviewed the camera trap images and captured “events” of animal appearances, using a 2‐min time interval as a cut off for a single event. We could not discern if the species in the images are multiple individuals or repeat individuals. We calculated detection rates by dividing the total number of sightings by the area of the camera (assuming a viewing frame of 1 m length and 0.5 m width), the number of cameras per week, and the number of weeks. The camera traps were unable to capture arthropods as the sensitivity was not capable of detecting the small body sizes and movement.

### 2.9 Data analysis

We used a generalized linear mixed effects model with a binomial distribution (link = logit) to analyze data collected of the prey models using the glmer function in the package lme4 (Bates et al., 2015). Predation (measured as the presence or absence of bite marks, measured after 7 days of deployment) was the response variable and color, patch pattern, color and patch pattern interaction, and month were included as fixed effects and each Virginia creeper cluster was run as a random effect nested into site. We also ran models where the patch pattern and color were considered as a single variable (red patterned, red solid, green patterned, green solid). Cluster nested into site was removed as a random effect when this variable did not account for the variance in the model (variance close or equal zero). Due to differences in color interpretation and hunting strategies by different predator taxa, we ran five variations on the model that either considered multiple taxa together, or separately: all predator taxa, arthropods only, vertebrates only, and separately by rodents and birds. Larger, non‐rodent mammals, although they had very distinctive bite marks, were not analyzed separately due to small sample size (*n* = 12). Additional separate models were run with predation as the response variable and treatment as the fixed variable. Afterward, we used the anova function to test the significance of the predictor variables in each generalized linear model and the mixed function in the afex package (Singmann et al., 2016) with a likelihood ratio test for the generalized linear mixed effects models. We then used Tukey's honest significant differences to perform post‐hoc pairwise comparisons for unequal sample sizes using the lsmeans package (Lenth, 2016) to determine pairwise significant differences.

In total, 24 prey models showed bite marks from two different predator taxa, and one prey model had bite marks from three different predator taxa. Therefore, to avoid artificially increasing the number of prey models that were used in our analysis, and due to the small sample size (2.6%, *n* = 25), we assigned numbers (1 & 2, or 1, 2 & 3) to the multiple identified predators on a singular prey model. We used a random number generator and the number the generator chose was kept and the other predator(s) were omitted. Thus, 25 predators were removed from the dataset of combined predators. The excluded predators were included in analyses including individual predator taxa (arthropods, birds, and rodents) because including the data would not inflate the number of prey models. To check if removing these data points would affect the results, we compared models with or without these data. When multiple predator data points were excluded, it resulted in one difference in the model outputs; significant predation on red prey models by rodents was lost. Randomly by chance, the excluded data points included predation by rodents on red models.

Additionally, we performed a chi‐squared test using raw counts of the data to test for non‐random associations between treatments and location of bite marks (anterior, middle, or posterior region of caterpillar prey model) received on the prey models by different predator taxa: arthropods, birds, and mammals (non‐rodent and rodent). All statistical analyses were performed in R software v. 4.1.2 (R Core Team, 2020) and R studio version 2022.02.0 + 443 (RStudio Team, 2020).

## 3 RESULTS

### 3.1 Weekly attack rates

Of the predated prey models (418/964), arthropods were responsible for 67.5% of attacks, birds 18.2%, rodents 11.5%, and large mammals 2.8%. These predation attempts expressed as an overall weekly attack rate for vertebrates and arthropods were 41.8% ± 2.3 (mean ± SE; bites prey model^−1^ week^−1^). Broken down by predator groups the rates were 28.2% ± 2.1 for arthropods, 7.6% ± 0.9 for birds, 4.8% ± 0.7 for rodents, and 1.2% ± 0.3 for large mammals. Attack rates were calculated from data excluding multiple predators.

### 3.2 Predation by prey model color, pattern, and treatment

**FIGURE 2:**
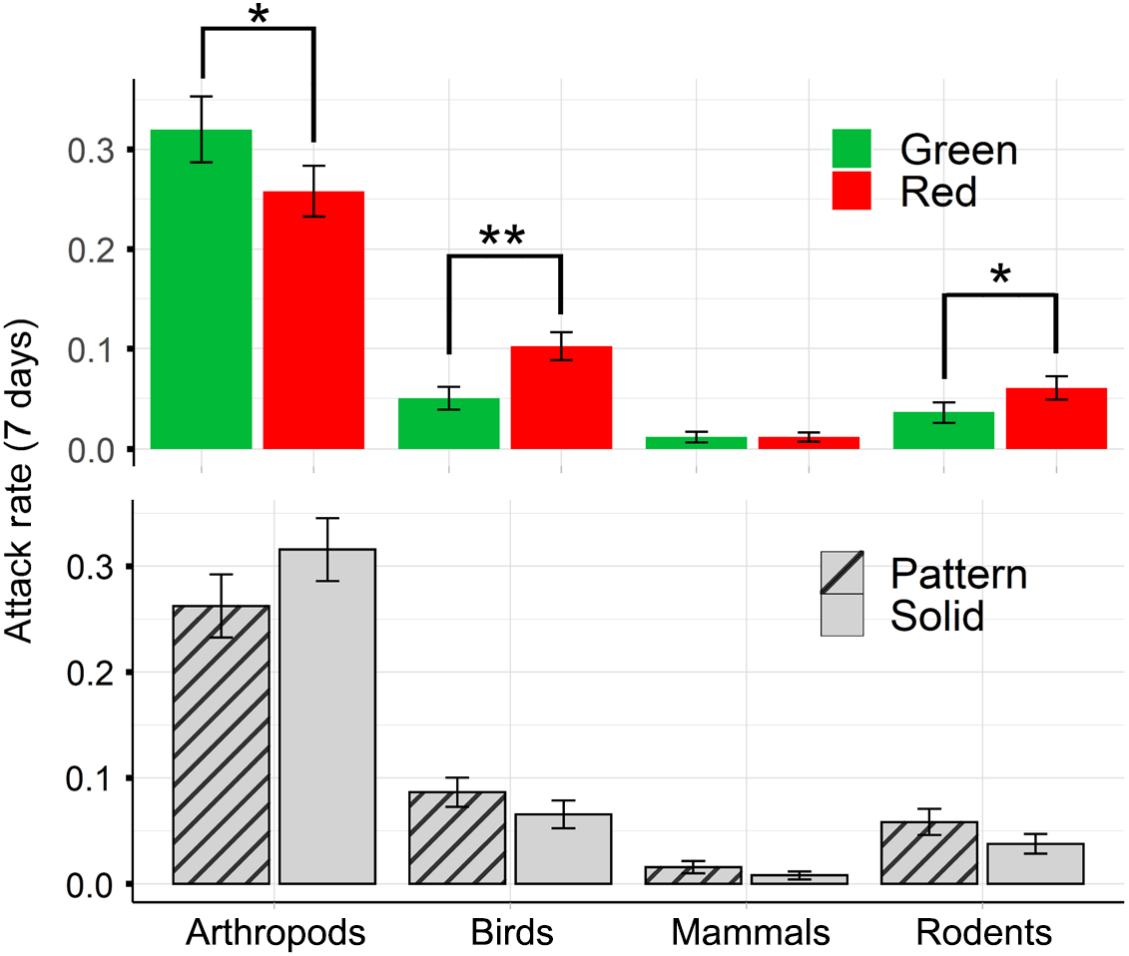
Predator attack rates by color and patch pattern. Attack rates (mean ± SE) on artificial prey models by color (top panel) and patch pattern (bottom panel) for predator groups averaged over all trials and sites. Birds and rodents had significantly higher predation on red than green prey models. Arthropods had significantly higher predation on green than red prey models (asterisks denote significance).

When all predators were analyzed together, color of the prey model, red or green, was not a significant predictor variable of predation (χ^2^ = 0.19, df = 1, *p* = .661). When analyzed separately, arthropod attack rates were significantly higher on green than red prey models (χ^2^ = 4.91, df = 1, *p* = .026; Figure 2). Whereas vertebrates (birds, large mammals, and rodents) had significantly higher attack rates on red than green prey models (χ^2^ = 6.03, df = 1, *p* = .014). When analyzed individually, both birds (χ^2^ = 7.57, df = 1, *p* = .006) and rodents (χ^2^ = 4.86, df = 1, *p* = .026) had significantly lower predation on green colored prey models than red (Figure 2).

The patterning, patch pattern versus solid (i.e., no patch), did not significantly influence predation (χ^2^ = 0.004, df = 1, *p* = .947) when vertebrates and arthropods were analyzed together. As with color, differences occurred when separate predator classes were considered. Arthropod predation did not vary significantly with patch patterning (χ^2^ = 1.10, df = 1, *p* = .295). However, vertebrates showed significantly higher predation on patch patterned over solid prey models (χ^2^ = 4.60, df = 1, *p* = .032; Figure 2). There were no significant interactions between color and patch patterning in any of our analyses.

**FIGURE 3:**
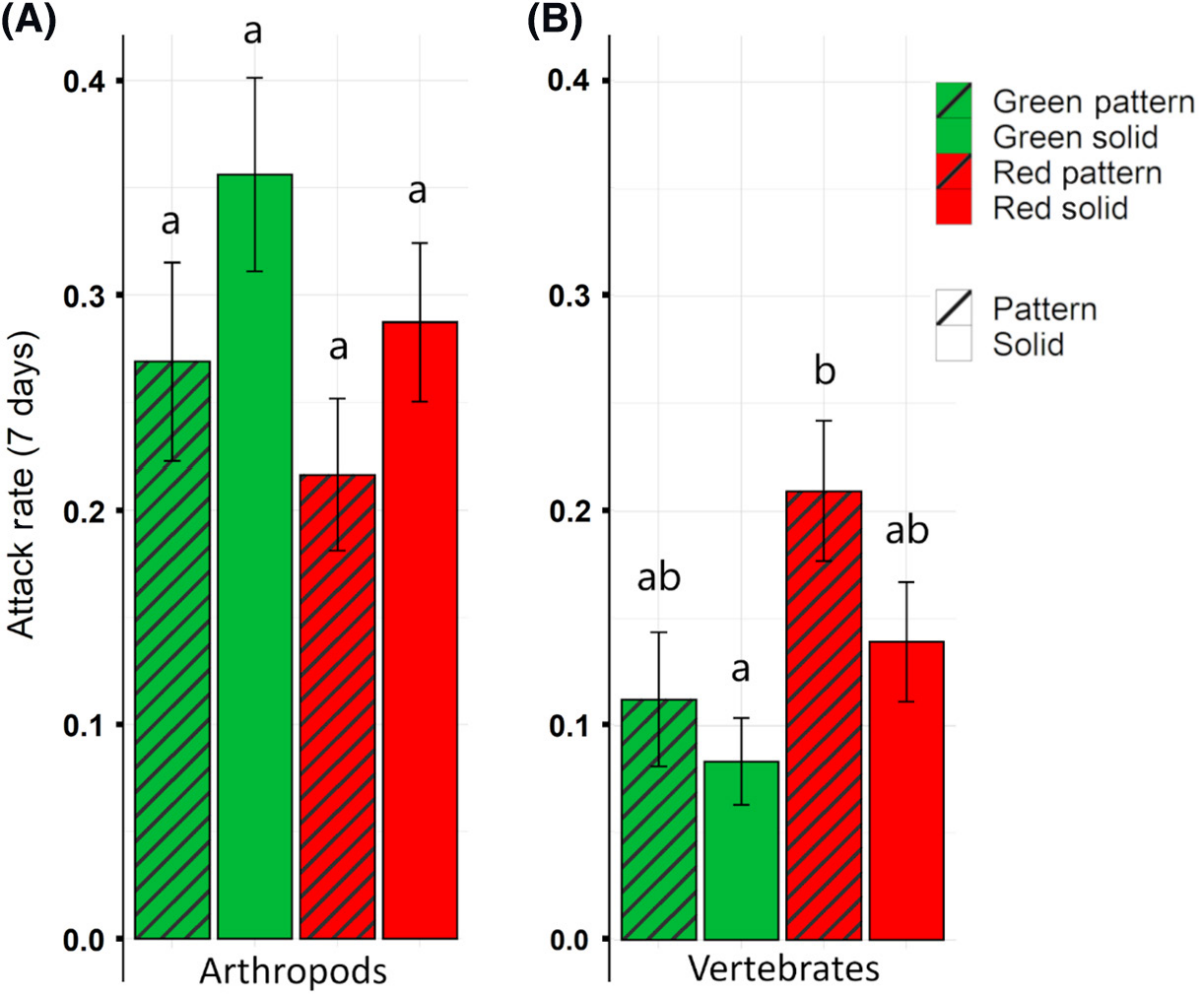
Arthropod and vertebrate attack rates by prey model treatment. Attack rates (mean ± SE) by arthropods (A) and vertebrates (B) on the four artificial prey model treatments (green pattern, green solid, red pattern, red solid) averaged over all trials and sites. Vertebrates had significantly higher attack rates on red patched prey models than on green solid prey models. Bars labeled with the same letters represent no significant statistical difference while bars with different letters represent statistically significant differences.

When considering the effect of color and pattern together, for vertebrates, prey model treatment influenced predation (χ^2^ = 11.56, df = 3, *p* = .009). In contrast, it did not affect arthropod predation (χ^2^ = 6.17, df = 3, *p* = .104; Figure 3). Vertebrates had significantly higher attack rates on red patched prey models in comparison to green solid prey models (Tukey's post hoc, *z* = −3.19, *p* = .008; Figure 3). Arthropods showed a tendency toward higher attack rates on green solid prey models than on red patched prey models, but non‐significant (Tukey's post hoc, *z* = 2.41, *p* = .076).

Additionally, the chi‐square tests suggest that there was no significant association between treatment and bite mark location on the prey models for arthropods (χ^2^ = 6.23, df = 6, *p* = .398), birds (χ^2^ = 9.49, df = 6, *p* = .147), and rodents (χ^2^ = 2.56, df = 6, *p* = .862). Non‐rodent mammal bite mark locations were not analyzed as the bites spanned the entire prey model, not a specific area.

### 3.3 Predation by season

**FIGURE 4:**
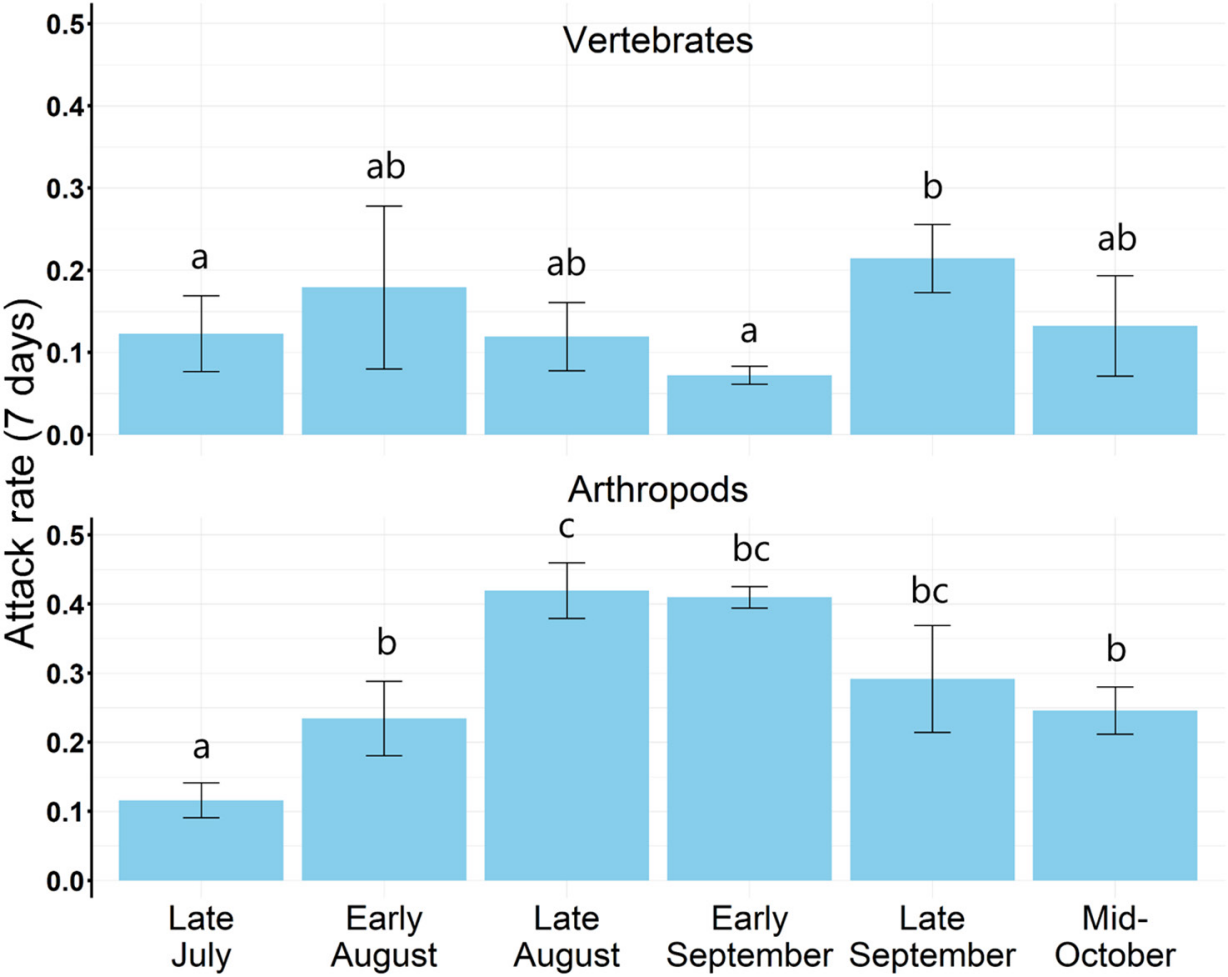
Arthropod and vertebrate attack rates by trial (right). (B) Attack rates (mean ± SE) across the six seasonal trials for vertebrates (top panel) and arthropods (bottom panel). Vertebrate attack rates did not show a clear seasonal trend but there was a significantly higher peak in late September (0.2 vs. 0.1 (bites prey model^−1^ week^−1^)) than in late July and early September. In contrast, arthropod attack rates peaked during late August.

Month was a significant predictor of predation attempts when all predators were analyzed together (*χ* ^2^ = 36.61, df = 5, *p* < .0001). For vertebrates, predation varied by month (*χ* ^2^ = 16.81, df = 5, *p* = .005). Specifically, vertebrate predation was significantly lower in early September (Tukey's post hoc, *z* = −3.20, *p* = .017) and late July (Tukey's post hoc, *z* = −3.04, *p* = .027) compared to late September (Figure 4). For rodents, month did not influence predation (*χ* ^2^ = 9.75, df = 5, *p* = .083) and bird predation only approached significance for lower predation in late July (*χ* ^2^ = 13.32, df = 5, *p* = .021, Tukey's post hoc, *z* = −2.64, *p* = .088) than late September. For arthropods, predation also varied by month (*χ* ^2^ = 49.20, df = 5, *p* < .0001). Arthropod predation was significantly lower in early August (Tukey's post hoc, *z* = −3.24, *p* = .015) and mid‐October (Tukey's post hoc, *z* = −3.18, *p* = .018) than in late August, and late July had fewer predation attempts by arthropods than any other period (all Tukey's post hoc with *p* values between .03 and <.0001; Appendix S1: Table S3 for exact values; Figure 4). There were no significant interactions between month and color in any of our analyses.

### 3.4 Pandora sphinx caterpillar color frequencies

**FIGURE 5:**
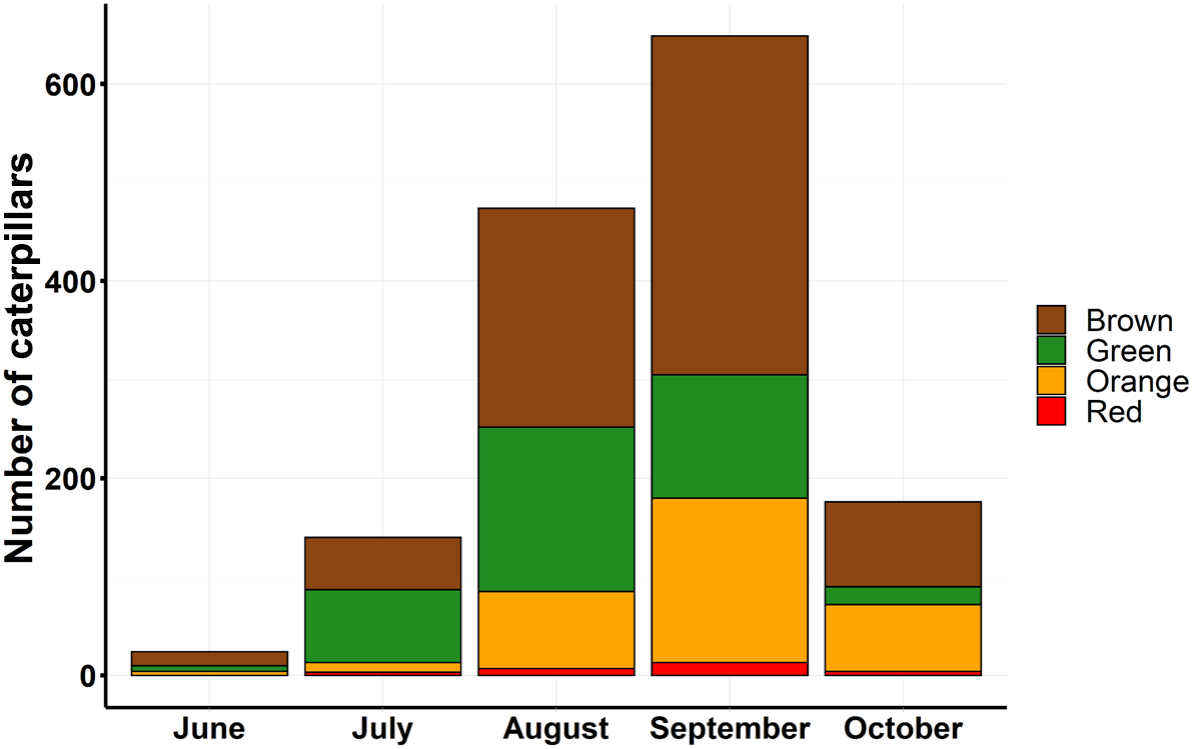
Pandora sphinx caterpillar colors observed by month. Data (*n* = 1478) were obtained from iNaturalist observations. Green proportions were highest in July. Brown, orange, and red proportions were highest in September.

Of the total 1478 Pandora sphinx caterpillar observations documented in iNaturalist, brown was the most frequent caterpillar color (49.2%), followed by green (26.7%), orange (22.2%), and last, red (1.8%). Fifth instar caterpillars were most frequently reported by observers (*n* = 1357). Brown, orange, and red colored caterpillars peaked in September and green colored caterpillars peaked in August (Figure 5). The multinomial logistic regression model predicted that proportions of green caterpillars will be highest in July and decrease as the season goes on. Brown caterpillar probability will peak in June, be lowest in July but increase as the season goes on with a slight decrease in October. Orange caterpillar probability will increase throughout the season and peak in October while red probability will have little variation throughout the season (Appendix S1: Figure S3). When plotting observations by day of year and latitude, it was evident that more observations have been reported at higher latitudes and that green and brown are more likely found at the beginning of the season compared with orange and red (Appendix S1: Figure S4). Given the nature of community science data, it is hard to discern if populations of the Pandora sphinx are denser in the northern region or if there are simply more observers in the north reporting on iNaturalist. The results of our chi‐squared test suggest that there is a relationship between color morph and month (*χ* ^2^ = 134.35, df = NA, *p*‐value = .0005; simulated *p*‐value based on 2000 replicates). All color morphs were more frequent in August and September than any other month.

### 3.5 Field estimates of densities of predators & prey

#### 3.5.1 Bird point counts

A total of 12 bird point counts (four at each site, 6 h total) were conducted in the habitats where prey models were deployed. The total number of insectivorous bird species documented were 14 at BH, 10 at GB, and 18 at BP (complete list of bird species observed, Appendix S1: Table S4). Of the 28 unique bird species, 22 were both insectivorous and ground foraging and after averaging all the bird point counts, it appeared that BP had both the highest number of species and abundance of birds (Appendix S1: Table S5).

#### 3.5.2 Camera traps

Throughout the duration of the study, 1726 trail camera images recorded 344 vertebrate encounters and one slug (Appendix S1: Table S6). Of all images captured, 322 contained potential predators (species with omnivorous diets) to the prey models. Among these were 10 species of birds and seven species of mammals (Appendix S1: Table S7). Of these, five (1.6%) animals were captured biting the prey models, one bird, an American robin (*Turdus migratorius*), during the daytime, and four mammals: a fisher (*Pekania pennanti*), also during the daytime; an unidentified mouse (Deer (*Peromyscus maniculatus*), White‐footed (*P. leucopus*), or Woodland jumping (*Napaeozapus insignis*)) during the night time; and two racoons (*Procyon lotor*), during the night time, while the rest appeared to be foraging or moving across the area. Based on these records we estimate vertebrate predator event detection rates were 10.8 vertebrate predators camera^−1^ week^−1^ and 0.2 vertebrate attacks camera^−1^ week^−1^. Discussion about the attack rates by site can be found in the Supplementary Materials.

## 4 DISCUSSION

Our study aimed to see how coloration and conspicuous patch patterning of prey model caterpillars affected predation and to gain insight into their potential anti‐predatory function. Our surveys and camera traps allowed us to investigate the array of visually oriented predators that forage at or near ground level that could be responsible for the attacks on caterpillar prey models. We found higher attack rates by birds and rodents on red colored prey models and higher attack rates on green prey models by arthropods. We hypothesized that the conspicuous patches on the Pandora sphinx caterpillar may function as an anti‐predatory visual defensive strategy following the distractive mark idea if we observed lower attack rates on green prey models with conspicuous patches. If there were lower attack rates on red prey models with patches, this would have supported avoidance by warning. Our results, however, did not support that the conspicuous patches found on the Pandora sphinx caterpillar function as distractive marks or as a warning. In contrast, we found a greater predation rate of red patched caterpillars by vertebrate predators. Our results suggest that the conspicuous patches and the red coloration by themselves might not be an adaptive trait to escape visual predators in some locations.

### 4.1 Attack rates as a function of taxon

Our daily attack rates were comparable to other attack rates reported in temperate forests (Bereczki et al., 2014; Lövei & Ferrante, 2017). However, Ferrante et al. (2017) found a much higher daily attack rate in forest fragments in Denmark for arthropods and vertebrates during the daytime (about 3× greater) and nighttime (almost 10× greater). Many factors can influence attack rates including varying predator preferences, type of habitat, size of habitat (Andrén et al., 1985), time of season, and degree of urbanization (Eötvös et al., 2018). Methodological variables that can affect attack rates include material used for artificial prey (Sam et al., 2015), and placement of the prey. For example, instead of using a natural substrate in the habitat, a few studies glued prey models to wooden or bamboo rods and then placed the rods into the soil (Ferrante et al., 2017; Frey et al., 2018; Kuli‐Révész et al., 2021). Our prey models were placed on naturally occurring host plants found within the habitats, following ecologically relevant placement. We did not target a specific predator taxon, but it is evident that placement of prey correlates to predation by specific predator types. Our attack rate results align with other studies that placed artificial prey at or near ground level and found that arthropod predators dominate despite the habitat (Ferrante et al., 2021; Lövei & Ferrante, 2017; Magagnoli et al., 2018). Arthropods emerged as the dominant predator even at 2 m above ground in habitats with various canopy covers (Seifert et al., 2015). Studies that placed artificial prey on leaves and branches of trees generally had higher bird predation (Bereczki et al., 2014; Hernández‐Agüero et al., 2020; Hossie & Sherratt, 2013), stressing how the placement of artificial prey can help target different types of predators.

### 4.2 Predation by prey model color, pattern, and treatment

Regarding color, we did not expect to see a difference in attack rate on red and green prey models because of varying detection and attraction factors. Post hoc photo analyses revealed that our prey model treatments did not have strong background matching in the field under human vision conditions but we can conclude that our green prey models were less conspicuous than our red prey models (Appendix S1: Table S1 & Figure S2). The color value (representing how ‘bright’ an object is) of the red prey models were significantly different from the leaf value, whereas the green prey models were not. However, the hue of green and red prey models were different from the leaves' hue (Appendix S1: Figure S7). Even though the green prey models could have been more conspicuous than we expected based on color analyses, we found that differences in attack rates were predator group specific; birds and rodents had higher attack rates on red than green prey models while arthropods had higher attack rates on green than red prey models. Because the biology and knowledge about each of these predator groups is varied, we discuss them separately below.

Day active birds are well‐known predators of caterpillars (for recent literature see Dekeukeleire et al., 2019; Zvereva et al., 2019) and their visual physiology allows them to see a wide range of the light spectrum (from UV into infrared) using three of four distinct light sensitive pigments (Bennett & Théry, 2007). They can easily detect red objects (Bennett & Théry, 2007) as demonstrated by the attraction toward many red fruits and berries that serve as a food source for birds (Lomascolo & Schaefer, 2010; Schmidt & Schaefer, 2004; Wheelwright, 1988) Despite this, red might also serve as a warning color indicating an unpalatable or poisonous caterpillar. Our results for birds support the findings by Hernández‐Agüero et al. (2020) suggesting that birds did not show aversion to red prey models. The avoidance of aposematic caterpillars usually involves learning and previous negative experience with unpalatable caterpillars displaying the warning signal (Joron, 2009). To our knowledge, there are no known red toxic caterpillars in Massachusetts (Wagner, 2005) so resident birds in our system may not have learned that red can serve as a warning color for caterpillar prey, potentially explaining the higher attack rates on red prey models, which could have been more easily detectable by birds.

Mammals, except primates (Peichl, 2005; Zhao et al., 2009), are generally thought to have limited color vision (Jacobs, 1993) because they possess only two cone types, making it difficult for them to distinguish between red and green colored objects. Instead, mammal eyes have evolved to see motion and contrast at lower light levels (Jacobs, 1993). In addition to vision, many mammals rely on olfaction for finding food (Hughes et al., 2010). Rodents showed significantly higher predation on red prey models in our study. We suspect that most of the bite marks on the prey models were from night active mice, not the day active chipmunks and gray squirrels, based on camera trap images. In a recent review, Leinonen and Tanila (2018) noted that the vision systems of rats and mice are adapted to detect motion and pattern at low dim light conditions but that some neurobiological information suggests they have better color vision than previously thought. Even if rodents cannot distinguish between the red and green prey models, the conspicuousness of the red prey models and increased contrast against the Virginia creeper plant compared to the green prey models' contrast could offer an explanation why rodents had a higher predation rate on red prey models. However, the significance was lost when we included multiple predation attempts per prey model in the statistical analysis. Thus, higher predation on red prey models by rodents is not robust.

Arthropods had significantly higher attack rates on green than red colored prey models. Our results are surprising given that arthropods rely more heavily on chemical cues than visual aspects regarding predation (Zvereva & Kozlov, 2016). For example, Aslam et al. (2020), found no significant differences by arthropod predators when presented with solid green, solid black, or black with aposematic coloration model prey. Nevertheless, a review studying predatory and parasitic arthropod vision systems concluded that many predatory and parasitic arthropods use a combination of vision and olfaction when hunting for prey. Although eye size varies greatly by species, those with larger, compound eyes may rely more heavily on visual cues and conversely, species with smaller eyes may rely more heavily on olfaction (Lim & Ben‐Yakir, 2020). Further, there are insect orders that are known to possess photoreceptor spectral sensitivity capabilities beyond 600 nm, the ability to see orange and red, which include Odonata, Hymenoptera, Coleoptera, and Lepidoptera (van der Kooi et al., 2021). As species of Hymenoptera (Hyodo et al., 2011) and Coleoptera (Becker, 1975) are known to have omnivorous diets, it is possible that these insects were able to distinguish between red and green prey models as potential predators at our sites. Based on the diversity of bite marks that we observed, it is clear that a heterogeneous group of arthropods were attacking the prey models. Further investigation of confirmed bite marks from insects representing each order is needed to support this. Our evaluation of chewing, scraping, gouging, or puncturing marks do not give us enough confidence to identify the level of the order of arthropods.

We recognize that there are limitations to the interpretation of attack rate by arthropods because they are more chemically oriented predators. It is possible that the arthropods did not perceive the prey models as caterpillars since they rely primarily on olfaction. A paper by Weissflog et al. (2022) examined attack rate on caterpillar shaped and humanoid shaped plasticine prey models and found that for invertebrates, shape was irrelevant. This emphasizes that further research is needed to assess the interpretation of artificial prey for non‐visual predators. Rather, the chemical properties of the plasticine may have served as an attractant to arthropod predators given that the prey model was novel within the forest habitat. Given these possible caveats, it is important to be cautious when interpreting arthropod attack rate data.

Regarding patch patterning, vertebrates (birds, larger mammals, and rodents) as a group showed higher predation on patch patterned than solid prey models but not when analyzed separately, each predator group only approached significance. Arthropods had a tendency of higher predation on solid colored prey models, although non‐significant. Although we did not test recognition times of predation by predators on our different treatments, if the distractive marks hypothesis had been supported, we would have expected to see significantly lower attacks on the green patch patterned treatments. Therefore, based on our results, we cannot provide support for the distractive marks hypothesis. In a study by Stevens, Marshall, et al. (2013), they found that distractive markings were either costly to survival or had neutral effects. Similarly, we did not find strong avoidance of patched prey models by predators in our study, and vertebrates may have had increased predation on patched prey models due to increased conspicuousness of the patches against the main body background color.

Contrary to our expectations, we found no significant interactions between color and patch patterning. Hossie and Sherratt (2012) found that eyespots and countershading, darker coloration dorsally and lighter coloration ventrally, on Canadian tiger swallowtail caterpillars (*Papilio canadensis*) interacted together to reduce predation. Based on these findings, we expected a similar interaction between coloration and patch patterning on Pandora sphinx caterpillars to reduce predation based on the distraction or warning hypotheses. However, our results did not support either of these hypotheses. Alternatively, it could be possible that coloration and/or patch patterning may interact with behavior, such as defensive posture (Hossie & Sherratt, 2013), or chemical cue to serve as an effective anti‐predatory mechanism. We acknowledge that, in order to isolate the effects of color and conspicuous patches, there are some physical traits that exist on live Pandora sphinx caterpillars that were excluded on our prey models, including a posterior anal horn and a posterior eyespot that is present once the horn falls off. Additionally, Pandora sphinx caterpillars have a defensive posture in which their head retracts into the anterior region, resulting in a swollen appearance (Wagner, 2005), which may contribute to predator avoidance behavior. This defensive posture could work in concert with the patches. Further investigation is warranted as the relationships between these traits and their interactions were beyond the scope of our study.

Further, our results could indicate that the patch patterning is not an anti‐predator trait, however, more investigation is needed. Caro and Allen (2017) offer that interspecific visual communication, in addition to anti‐predatory, could also serve other functions, such as food acquisition, anti‐parasite, host acquisition, and agonistic signaling. In respect to the conspicuous, oval shaped patches that are found on Pandora sphinx caterpillars, it is more likely that they could serve as anti‐predatory or anti‐parasitoid function. The idea of an anti‐parasitoid function assumes that potential parasitoids use visual cues when selecting a host, such as the presence of other parasitoids that could serve as competitors (Stireman & Shaw, 2022). Parasitoids, such as *Cotesia congregata* (Hymenoptera: Braconidae), a common parasitoid wasp of Pandora sphinx, lay their eggs in caterpillar hosts the larvae exit the host by spinning cream colored oval shaped cocoons attached to the caterpillar's integument (Lampert et al., 2010). The patches found on Pandora sphinx caterpillars could resemble these cocoons, but the potential of these patches serving as anti‐parasitoid function has yet to be tested. Alternatively, the patch patterning might not be an adaptive trait. Similar to the eyespots in *Eumorpha* (Ponce et al., 2015), the lateral patches may simply have been conserved through their evolutionary history and might not be adaptive, suggesting that they may not provide benefits with respect to survival. Although there is no current evidence to support this idea, further genetic work is needed to elucidate the evolution of patch patterning in *Eumorpha*.

### 4.3 Predation by season

Seasonality can play a role in predator–prey interactions and the effectiveness of defensive strategies due to predator composition and diet preferences as many predators can vary diet based on season and when food resources are available (Carvalho et al., 2019; Richards & Coley, 2007; Schmidt et al., 2008). Contrary to our predictions, we observed a seasonal trend in arthropod predation, but not among birds or mammal predators. These results were not expected as we assumed the attack rates would be highest in late July due to fledgling birds increasing predation pressure, but it is possible that alternative food resources were available (Moser et al., 2006; Richards & Coley, 2007), as many predators in our system were omnivorous. Our results varied slightly from findings in a forest in Denmark where chewing insect attack rates were lowest in spring and highest in autumn (Ferrante et al., 2014). In our study, the arthropods had the lowest predation at the end of July and predation peaked in late August and early September, which is approaching the end of the summer. Predation decreased in late September and mid‐October, early fall, but rates were still higher than in late July. A field experiment conducted in Finland by Mappes et al. (2014) concluded that seasonality of predator composition can impact the effectiveness of cryptic versus conspicuous strategy. They found that during the time of fledging for young birds, the protective value of cryptic coloration increased, and the protective value of conspicuous coloration decreased due to naivety. The opposite trend was found later in the season when fledglings were rare. However, in our study, we did not find variation in predation by birds based on season, potentially because of the lack of aversion to red coloration in our sites.

### 4.4 Pandora sphinx caterpillar color frequencies

The most common color morph for late instars of Pandora sphinx was brown (49.2%), followed by green (26.7%) and orange (22.2%). Green and brown morphs are broadly understood to match the background colors of the plants on which the caterpillars eat and rest. The red color morph was much rarer (1.8%). Regarding color morph frequency in populations, Johannesson and Butlin (2017) investigated wild snails and suggested that multiple mechanisms may be at play to maintain rare and conspicuous colors within a population. They investigated drift, migration, directional selection, heterozygote advantage, and frequency‐dependent selection but found that heterozygote advantage and negative frequency dependence were most explanatory. Pandora sphinx caterpillars are palatable, and the potential for the red coloration to function as a warning signal relies on these palatable caterpillars to be at lower density than other red unpalatable caterpillars (i.e., Batesian mimicry (Huheey, 1976)). In Massachusetts, red unpalatable caterpillars are absent, thus it is unlikely that Batesian mimicry is at play. We cannot comment on the mechanisms that maintain Pandora sphinx caterpillar morphs as further research is needed. However, these mechanisms could help explain why red morphs still exist in Pandora sphinx populations, although rare. Our data suggest that the non‐green morphs are more common later in the season, closer to the transition from summer to fall when it is more likely that Virginia creeper host plants might be drying up and changing to orange and red colors (Ip et al., 2010). Interestingly, we found that Pandora sphinx caterpillar color morph is dependent on month. It is known that host plant compounds can influence caterpillar color (Fink, 1995; Greene, 1989). In turn, this supports the idea of background matching where caterpillars are more cryptic if they resemble the hues of their host plant. Therefore, the non‐green color morphs that occur later in the season may in fact appear cryptic to predators against red and orange hued Virginia creeper, emphasizing that background matching is dependent on season. Wennersten and Forsman (2009) found that red colored artificial prey had the lowest survival rate and were the first to go “extinct” in both polymorphic and monomorphic populations. The frequencies of red caterpillars in the wild align with our results of red prey models being favored by vertebrate predators.

## 5 CONCLUSION

We found that more visually oriented vertebrates showed significantly higher attack rates on red and patch patterned prey models than on green solid prey models, while more chemically oriented arthropods had significantly higher attack rates on green prey models with a tendency for higher attack rates on solid prey models, though non‐significant. Therefore, our results do not support conspicuous patch patterning on the Pandora sphinx as an anti‐predatory trait under the idea that they serve as distraction marks or warnings to potential predators. Thus, patch patterning may not be associated with predation avoidance. Even though we did not find evidence that patch patterning decreases predation, seasonal differences in attack rates by arthropods suggest that Pandora sphinx caterpillar patch patterning function could be context‐dependent. Furthermore, the conspicuous patches found on this species may work in combination with a behavioral or chemical mechanism to help deter predation from visual predators. When thinking about the roles of visual or behavioral defenses employed by caterpillars, it is important to remember that certain defensive strategies may be more successful toward different predators and parasite taxa. Our findings demonstrate that further research is needed to explore the functionality, if any, and evolution of conspicuous patch patterning.

## AUTHOR CONTRIBUTIONS

**Julia Barrone:** Conceptualization (lead); data curation (lead); formal analysis (lead); funding acquisition (equal); investigation (lead); methodology (equal); project administration (lead); resources (equal); supervision (equal); validation (equal); visualization (equal); writing – original draft (equal); writing – review and editing (equal). **Mayra C. Vidal:** Conceptualization (supporting); data curation (supporting); formal analysis (equal); methodology (equal); supervision (equal); validation (supporting); visualization (equal); writing – review and editing (equal). **Robert Stevenson:** Conceptualization (supporting); data curation (equal); formal analysis (equal); funding acquisition (equal); investigation (supporting); methodology (equal); project administration (supporting); resources (equal); supervision (equal); validation (equal); visualization (equal); writing – original draft (equal); writing – review and editing (equal).

## CONFLICT OF INTEREST STATEMENT

We confirm that this work is original and has not been published elsewhere, nor is it currently under consideration for publication elsewhere. We declare no conflicts of interest.

## FUNDING INFORMATION

Funding was provided by the Biology Department at the University of Massachusetts, Boston and by efforts made through experiment.com.

### OPEN RESEARCH BADGES

This article has earned Open Data and Open Materials badges. Data and materials are available at https://github.com/jbarrone/Sphingid_caterpillars_conspicuous_patches.

## Supporting information


Data S1

## Data Availability

Data for the project is stored publicly here: https://github.com/jbarrone/Sphingid_caterpillars_conspicuous_patches DOI: https://zenodo.org/record/8124816.
