# Supplementary material for: Sphingid caterpillars conspicuous patches do not function as distractive marks or warning against predators
Source: Ecol Evol. 2023 Jul 23;13(7):e10334. doi: 10.1002/ece3.10334 (PMC10363802; doi:10.1002/ece3.10334)
Supplement: Supplementary file 1 — Data S1 [file ECE3-13-e10334-s001.docx]

Sphingid caterpillars conspicuous patches do not function as distractive marks or warning against predators

Julia Barrone | Mayra C. Vidal | Robert Stevenson

**Supplementary Material**

**Table S1. Values of color overlap of each model treatment against the field background under human viewing conditions (i.e., greater values, more overlap).** Although there was very little overlap with our models and the field background, our green models are more closely matched to the field background than the red models, which was expected. We acknowledge that bird, mammal and arthropod vision capabilities differ from that of humans, however, as our camara did not allow for UV capture, we were not able to consider bird vision. The field photos for the analysis were taken in early June which is not an accurate representation of the months in which we conducted our trials (late July through mid-October). Forest ground foliage is known to vary throughout the seasons, especially Virginia creeper leaf color.

|  | **Green** | **Green Solid** | **Red** | **Red Solid** |
| --- | --- | --- | --- | --- |
| Field Background | 0.01 | 4.234E-4 | 0 | 7.227E-6 |
|  |  |  |  |  |

**Table S2. List of Lepidoptera caterpillar species found during the night surveys.** A total of 6 nocturnal surveys were conducted at the sites between 10 pm-12 am from late August until late September.

| **Site** | **Date** | **Species** | **Count** |
| --- | --- | --- | --- |
| GB | 8/24 | Walnut Sphinx (*Amorpha juglandis)* | 1 |
| GB | 8/26 | Noctuidae spp. | 1 |
| BH | 8/26 | Huckleberry Sphinx (*Paonias astylus)* | 1 |
| BH | 8/26 | Lymantriinae spp. | 1 |
| BH | 8/26 | Limacodidae spp. | 2 |
| BP | 8/30 | Lymantriinae spp. | 1 |
| GB | 9/8 | Purple-crested Slug Moth *(Adoneta spinuloides)* | 1 |
| BH | 9/11 | Huckleberry Sphinx (*Paonias astylus)* | 1 |
| BH | 9/11 | Limacodidae spp. | 1 |

**Table S3. Output from Tukey’s post hoc test regarding arthropod predation by month.** Late July had fewer predation attempts by arthropods than any other period.

| **Month Comparison** |  | Estimate | SE | Z ratio | P value |
| --- | --- | --- | --- | --- | --- |
| early August - late July |  | 1.0073 | 0.337 | 2.993 | 0.0329 |
| early September - late July |  | 1.6844 | 0.323 | 2.993 | <.0001 |
| early September - late July |  | 1.8222 | 0.324 | 5.616 | <.0001 |
| late July - late September |  | -1.4404 | 0.330 | -4.366 | 0.0002 |
| late July - mid October |  | -1.0560 | 0.332 | -3.185 | 0.0182 |

**Table S4. Complete list of bird species documented during point counts.** A total of 12 bird point counts (4 at each site) were conducted from July through October.

|  |  | **Site** |  |  |
| --- | --- | --- | --- | --- |
| **Species** | **BH** | **BP** | **GB** | **Total** |
| American Crow |  | 1 |  | 1 |
| American Goldfinch |  | 2 |  | 2 |
| American Robin | 1 |  |  | 1 |
| Black-capped Chickadee | 3 | 1 | 4 | 8 |
| Blue Jay |  | 4 | 3 | 7 |
| Brown Creeper | 1 |  | 1 | 2 |
| Carolina Wren |  | 1 | 1 | 2 |
| Common Grackle | 1 | 1 |  | 2 |
| Common Yellowthroat Warbler | 1 | 2 |  | 3 |
| Downy Woodpecker | 1 |  | 2 | 3 |
| Eastern Towhee | 2 | 3 |  | 5 |
| Eastern Wood Pewee | 3 | 2 | 2 | 7 |
| Gray catbird |  | 1 | 1 | 2 |
| Hairy Woodpecker |  | 1 | 1 | 2 |
| Northern Cardinal | 1 | 1 |  | 2 |
| Northern Flickr | 1 | 1 |  | 2 |
| Ovenbird | 1 |  |  | 1 |
| Pileated Woodpecker |  | 2 | 1 | 3 |
| Red-bellied Woodpecker | 1 | 3 | 2 | 6 |
| Red-breasted Nuthatch | 1 | 1 |  | 2 |
| Red-eyed Vireo | 2 | 1 |  | 3 |
| Scarlet Tanager |  |  | 1 | 1 |
| Swallow sp. |  |  | 1 | 1 |
| Tufted Titmouse | 3 | 4 | 1 | 8 |
| White-breasted Nuthatch | 2 | 3 | 4 | 9 |
| White-throated Sparrow |  | 1 |  | 1 |
| Wood Thrush |  | 1 |  | 1 |
| Yellow-throated Vireo |  | 1 |  | 1 |
| **Total** | **25** | **38** | **25** | **88** |

**Table S5. List of insectivorous bird species from point counts and mean numbers of bird species and individual birds.** Raw counts of insectivorous bird species from the point count surveys at each site (top) and number of bird species and birds (expressed as mean ± SE; bottom) at each site, averaged across bird point counts transects conducted from July through October. n=4 observations per each site, 3 point counts per each transect.

|  |  | **Site** |  |  |
| --- | --- | --- | --- | --- |
| **Insectivorous Species** | **BH** | **BP** | **GB** | **Total** |
| American Robin | 1 |  |  | 1 |
| Blue Jay |  | 4 | 3 | 7 |
| Carolina Wren |  | 1 | 1 | 2 |
| Common Grackle | 1 | 1 |  | 2 |
| Common Yellowthroat | 1 | 2 |  | 3 |
| Downy Woodpecker | 1 |  | 2 | 3 |
| Eastern Towhee | 2 | 3 |  | 5 |
| Eastern Wood-Pewee | 3 | 2 | 2 | 7 |
| Gray Catbird |  | 1 | 1 | 2 |
| Hairy Woodpecker |  | 1 | 1 | 2 |
| Northern Cardinal | 1 | 1 |  | 2 |
| Northern Flicker | 1 | 1 |  | 2 |
| Ovenbird | 1 |  |  | 1 |
| Red-bellied Woodpecker | 1 | 3 | 2 | 6 |
| Red-breasted Nuthatch | 1 | 1 |  | 2 |
| Red-eyed Vireo | 2 | 1 |  | 3 |
| Scarlet Tanager |  |  | 1 | 1 |
| Tufted Titmouse | 3 | 4 | 1 | 8 |
| White-breasted Nuthatch | 2 | 3 | 4 | 9 |
| White-throated Sparrow |  | 1 |  | 1 |
| Wood Thrush |  | 1 |  | 1 |
| Yellow-throated Vireo |  | 1 |  | 1 |
| **Total** | **21** | **32** | **18** | **71** |
| Number of species | 5.3 ± 0.63 | 8.5 ± 0.41 | 4.5 ± 0.96 |  |
| Number of birds | 10.7 ± 1.3 | 14.8 ± 2.0 | 8.8 ± 2.4 |  |

**Table S6. Complete list of animal encounters captured on the trail cameras.** Camera trap records of 3 cameras placed at BP and 2 at GB. Each camera was deployed at one plot, focused on one model for the entire week.

| **Animal** | **BP** | **GB** | **Total** |
| --- | --- | --- | --- |
| American Robin | 5 | 1 | 6 |
| American Woodcock | 1 | 0 | 1 |
| Bird | 0 | 1 | 1 |
| Blue Jay | 3 | 1 | 4 |
| Deer/White-footed/Woodland jumping Mouse | 76 | 16 | 92 |
| Downy Woodpecker | 2 | 0 | 2 |
| Eastern Chipmunk | 45 | 37 | 82 |
| Eastern Gray Squirrel | 10 | 86 | 96 |
| Eastern Towhee | 1 | 0 | 1 |
| Fisher | 2 | 0 | 2 |
| Gray Catbird | 1 | 0 | 1 |
| Large Mammal | 4 | 2 | 6 |
| Northern Flicker | 1 | 0 | 1 |
| Ovenbird | 3 | 1 | 4 |
| Raccoon | 4 | 1 | 5 |
| Ruby-crowned Kinglet | 0 | 1 | 1 |
| Slug | 1 | 0 | 1 |
| Southern Flying Squirrel | 4 | 1 | 5 |
| Tufted Titmouse | 4 | 1 | 5 |
| Mustelinae spp. | 1 | 0 | 1 |
| White-breasted Nuthatch | 2 | 0 | 2 |
| White-tailed Deer | 11 | 2 | 13 |
| Wood Thrush | 12 | 1 | 13 |
| **Total** | **193** | **152** | **345** |

**Table S7.** **List of potential predators of the prey models, with omnivorous diets, captured on the trail cameras.** There were 3 trail cameras placed at BP and 2 at GB. Each camera was deployed at one plot, focused on one model for the entire week. To compare rates, the records at GB need to be multiplied by 1.5.

| **Bird Species** | **BP** | **GB** | **Mammal Species** | **BP** | **GB** |
| --- | --- | --- | --- | --- | --- |
| American Robin | 5 | 1 | Deer Mouse | 76 | 16 |
| Blue Jay | 3 | 1 | Eastern Chipmunk | 45 | 37 |
| Downy Woodpecker | 2 |  | Eastern Gray Squirrel | 10 | 86 |
| Eastern Towhee | 1 |  | Fisher | 2 |  |
| Gray Catbird | 1 |  | Raccoon | 4 | 1 |
| Northern Flicker | 1 |  | Southern Flying Squirrel | 4 | 1 |
| Ovenbird | 3 | 1 | Weasel | 1 |  |
| Tufted Titmouse | 4 | 1 |  |  |  |
| White-breasted Nuthatch | 2 |  |  |  |  |
| Wood Thrush | 12 | 1 |  |  |  |
| **Bird Totals** | **36** | **5** | **Mammal Totals** | **142** | **141** |

**Table S8. Regression coefficients from the multinomial logistic regression**. Table shows the regression coefficients ± SE and Wald statistics. Brown and June were used as base color and month in the model.

|  |  |  |  |  | **Month** |  |
| --- | --- | --- | --- | --- | --- | --- |
| **Color** | **Intercept** |  | **July** | **August** | **September** | **October** |
| Green | -0.85 ± 0.488,  z= -1.74,  p=.08 |  | 1.18 ± 0.520,  z= 2.27,  p=.02 | 0.56  ± 0.499,  z= 1.13,  p=.26 | -0.16 ± 0.499,  z= -0.33,  p=.74 | -0.72 ± 0.553,  z= -1.30,  p=.19 |
| Orange | -1.25 ± 0.567,  z= -2.21,  p=.03 |  | -0.41 ± 0.664,  z= -0.62,  p=.53 | 0.21 ± 0.582,  z= 0.36,  p=.72 | 0.53 ± 0.575,  z= 0.92,  p=.36 | 1.02 ± 0.590,  z= 1.73,  p=.08 |
| Red | -11.92  ± 103.477,  z= -0.12,  p=.91 |  | 9.05 ± 103.479,  z= 0.09,  p=.93 | 8.46 ± 103.478,  z= 0.08,  p=.93 | 8.64 ± 103.478,  z= 0.08,  p=.93 | 8.85 ± 103.479,  z= 0.09,  p=.93 |


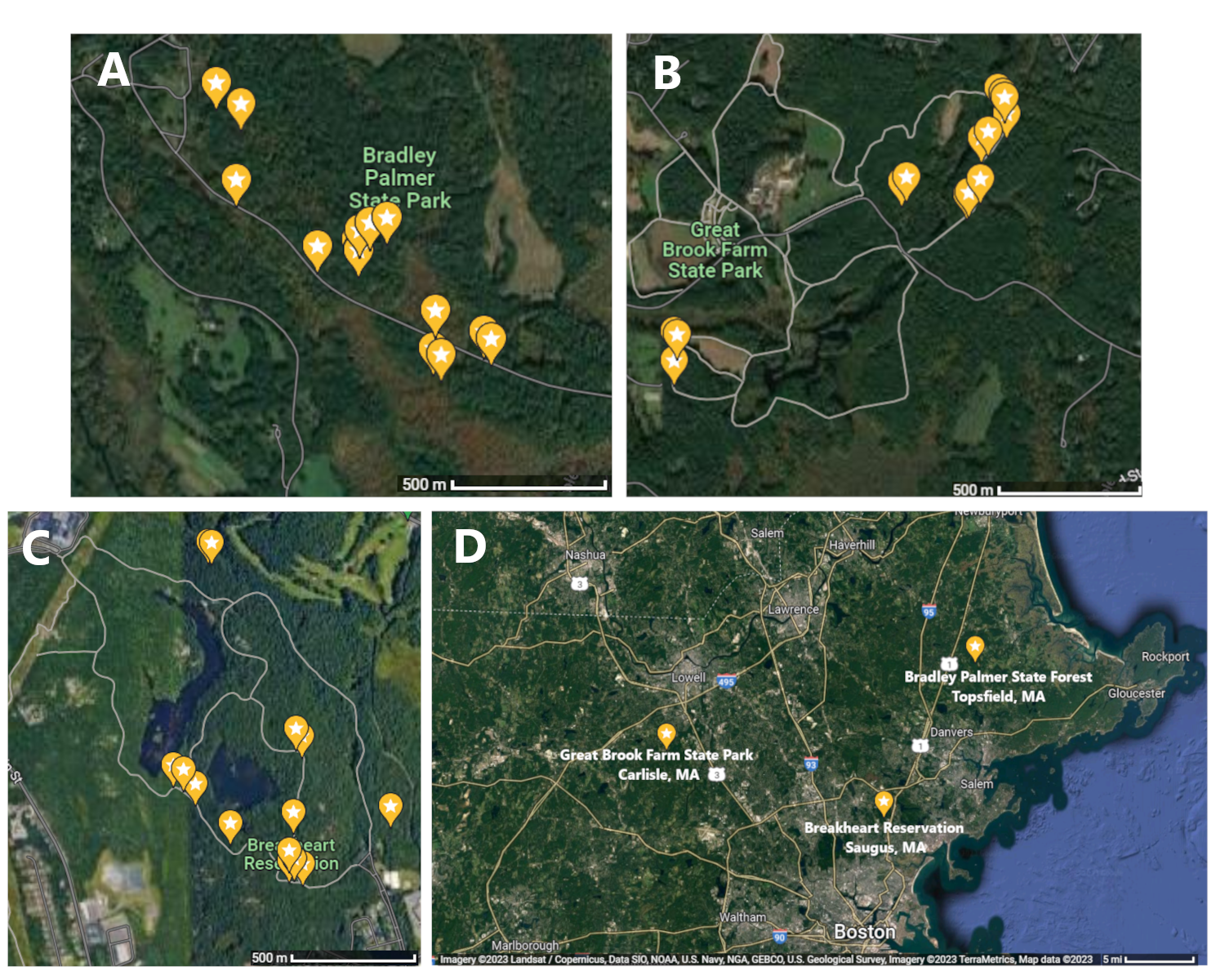


**Figure S1.** **Satellite images of the three sites in Massachusetts**. A) Virginia creeper cluster locations at Bradley Palmer State Park. B) Cluster locations at Great Brook Farm State Park. C) Cluster locations at Breakheart Reservation. D) Map depicting all three sites.


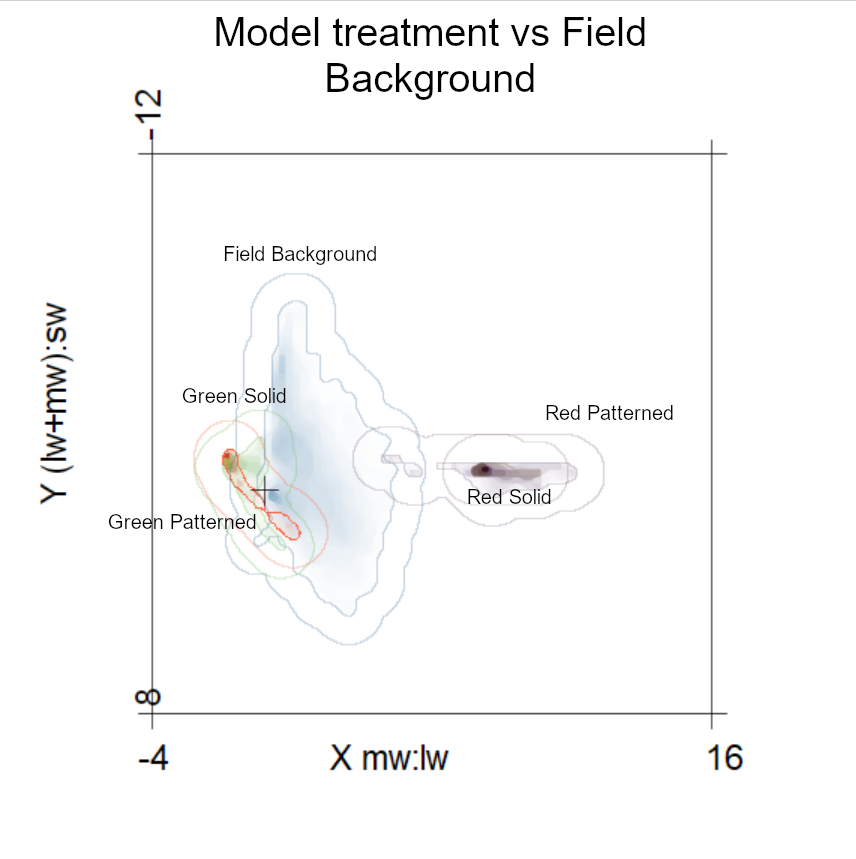


**Figure S2.** **Color map images of each model treatment against the field background under human viewing conditions.** The color maps show the degree to which there is color similarity. The field background is indicated by the blue color, green solid model by the green color, green patch patterned model by the red, red solid model by the brown and red patch patterned model by the purple color in the map. Although there was very little overlap with our models and the field background, our green models are more closely matched to the field background than the red models, which was expected. SW, MW and LW stand for short, medium and long wavelengths.


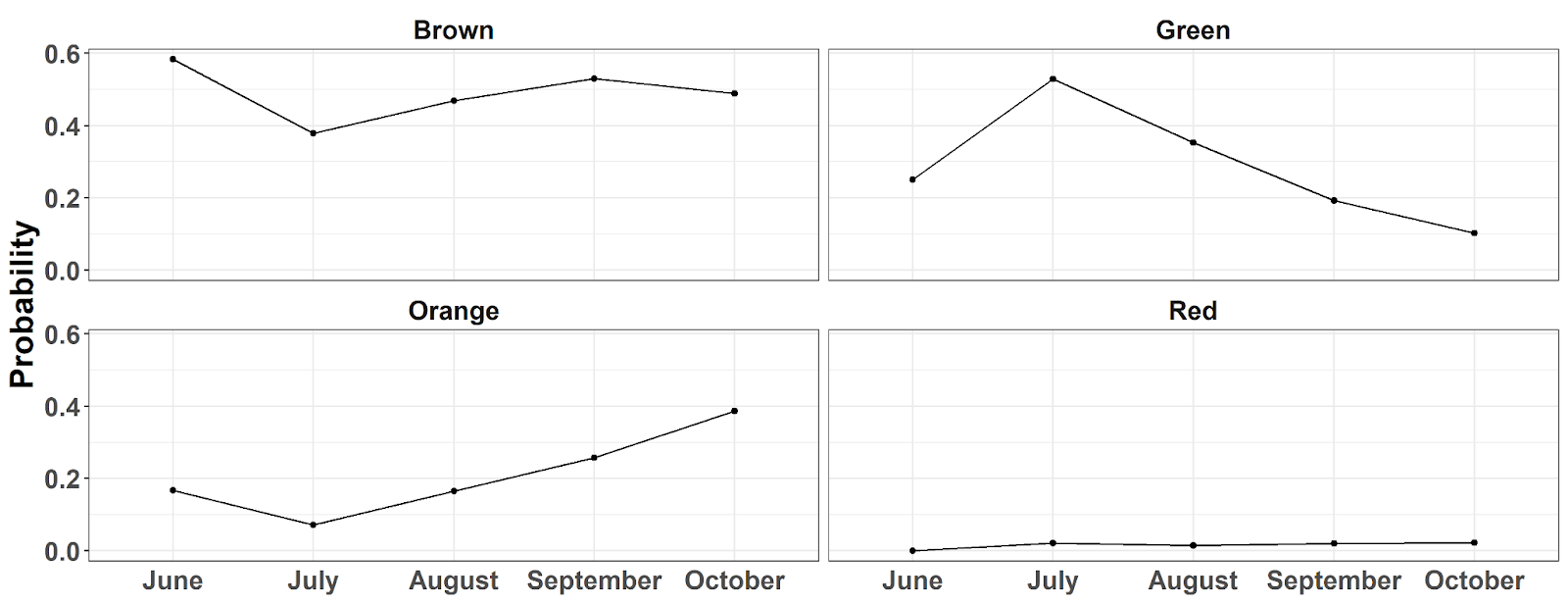


**Figure S3. Predicted probabilities of larvae color by month.** Probabilities were generated from the multinomial logistic regression using the data from iNaturalist. The data was filtered to only include observations from June - October. The highest probability of being green is in July, brown in June and orange in October. Red is constant.


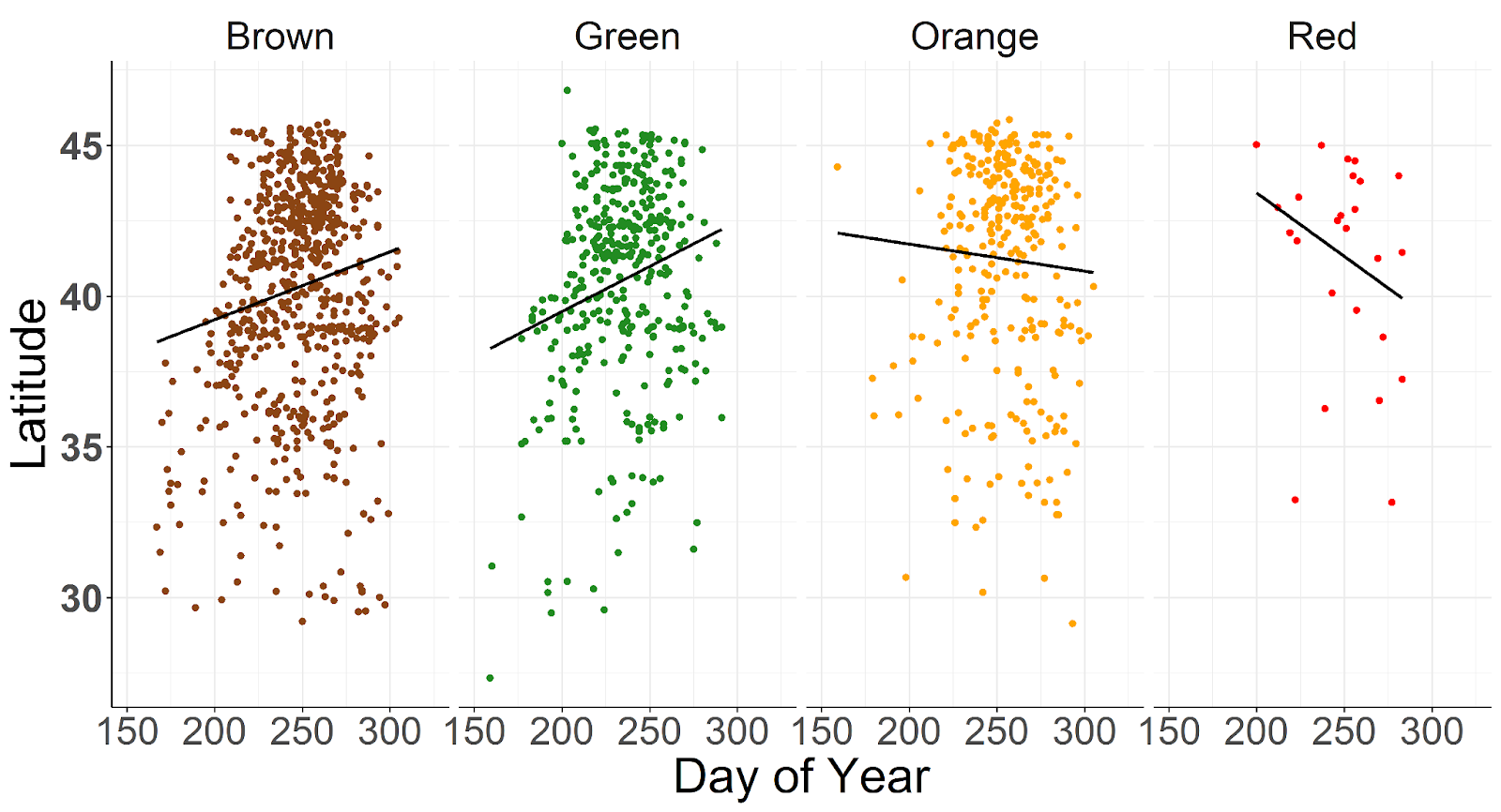


**Figure S4. Observations of Pandora sphinx caterpillar colors by day of the year and latitude.** Data was obtained from iNaturalist observations and has been filtered to show observations from June - October. Latitude ranged from 27.33 to 46.83. June 1st is day 152 and November 16th is 320. Sample sizes are given in figure S3. Trendline shows the mean.


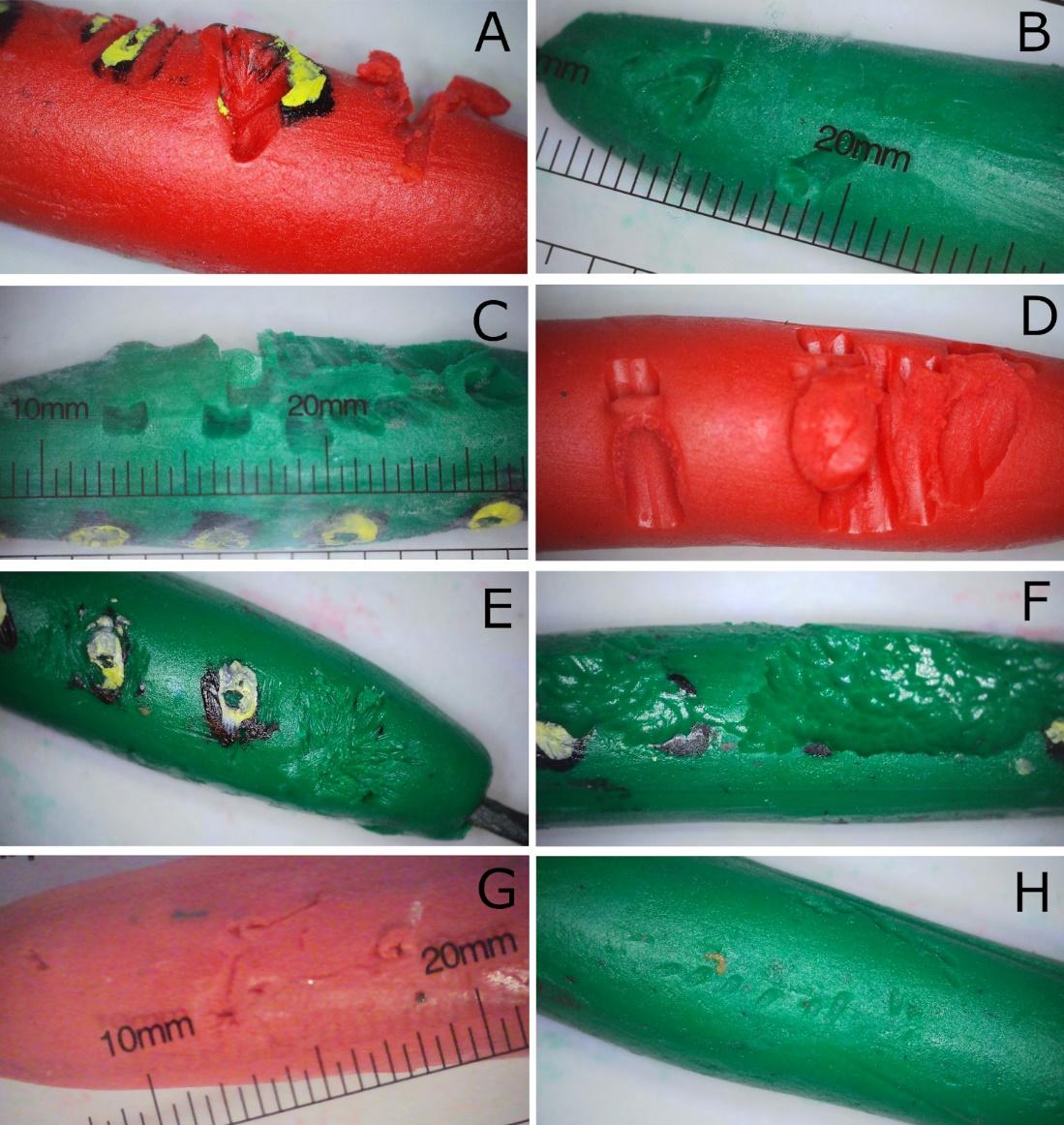


**Figure S5.** **Attacked artificial models showing predation marks from a variety of predators**. Predator key: A-B: avian, C-D: rodent, E-H: arthropod. Images taken with a digital microscope (Bysameyee HD 2MP USB). See figure S5 for an example of marks made by a larger mammal.

**
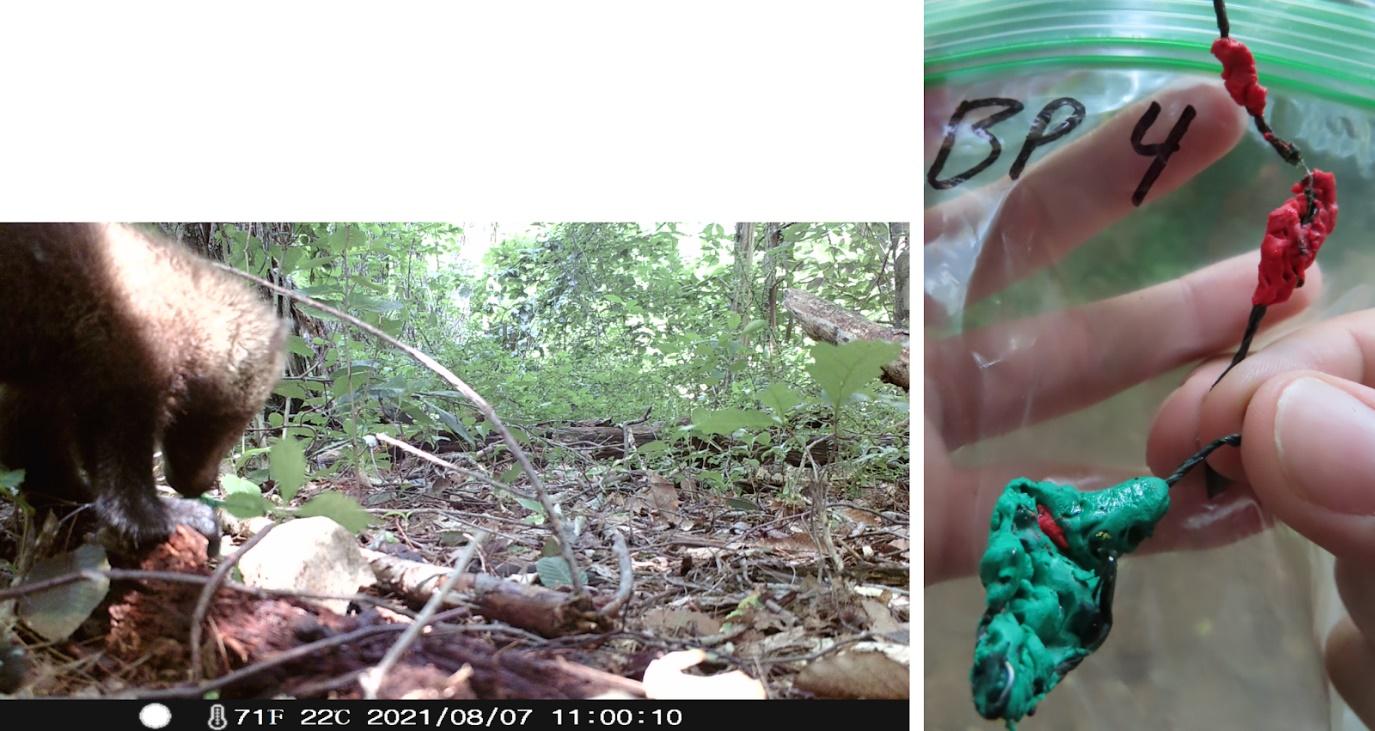
**

**Figure S6. Trail camera image and photograph of recovered prey models.** Trail camera image from BP depicting a fisher about to predate a green solid model (left). The recovered red solid and green solid models bitten by the fisher (right).

 
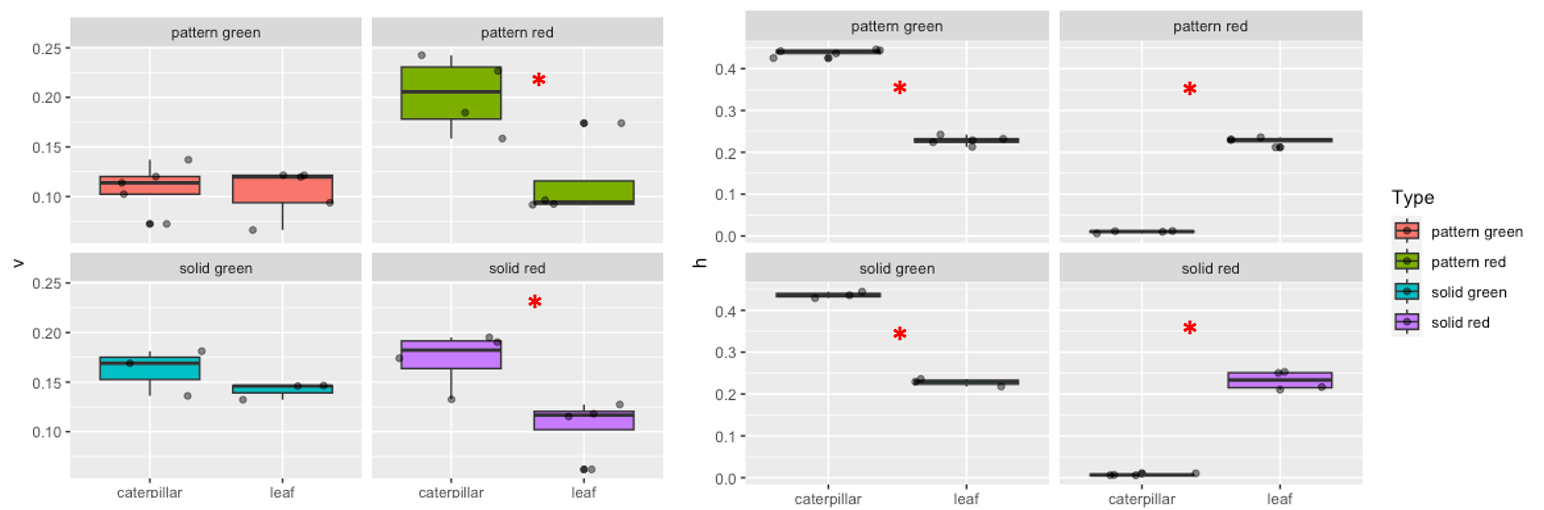


**Figure S7:** **Differences of value (‘v’, left panels) and hue (‘h’) between caterpillar plasticine models of each type and the Virginia Creep leaf.** Analyses were performed by extracting the RGB values from regions of interest using MICA toolbox, converting RGV to HSV using the function in R, and conducting an ANOVA with h or v as the response variable and the type of plasticine model as the fixed effect, followed by Tukey’s pairwise comparisons. Red asterisks show significant pairwise differences.

**Predation by Site**

To test for the potential difference in predation rate by site, we performed a generalized linear mixed effects model with a binomial distribution (link=logit) to analyze data collected of the prey models using the glmer function in the package lme4 (Bates *et al.,* 2015). Predation was the response variable and color, patch pattern, color and patch pattern interaction, month, and site were included as fixed effects and cluster number was run as a random effect.

Site was a significant factor influencing predation when all predators were analyzed together (χ^2^=16.44, df=2, p<0.001). All predators together showed significantly lower predation at BH (Tukey's post hoc, z=-2.86, p=0.012) than BP and significantly higher predation at BP (Tukey's post hoc, z=3.88, p<0.001) than GB. This result was mainly driven by vertebrates as a group (χ^2^=22.44, df=2, p<0.001), which showed significantly higher predation at BP compared to GB (Tukey's post hoc, z=3.88, p<0.001) and BH (Tukey's post hoc, z=-3.92, p<0.001) . Conversely, arthropod predation showed no significant differences among sites (χ^2^=2.26, df=2, p=0.323).


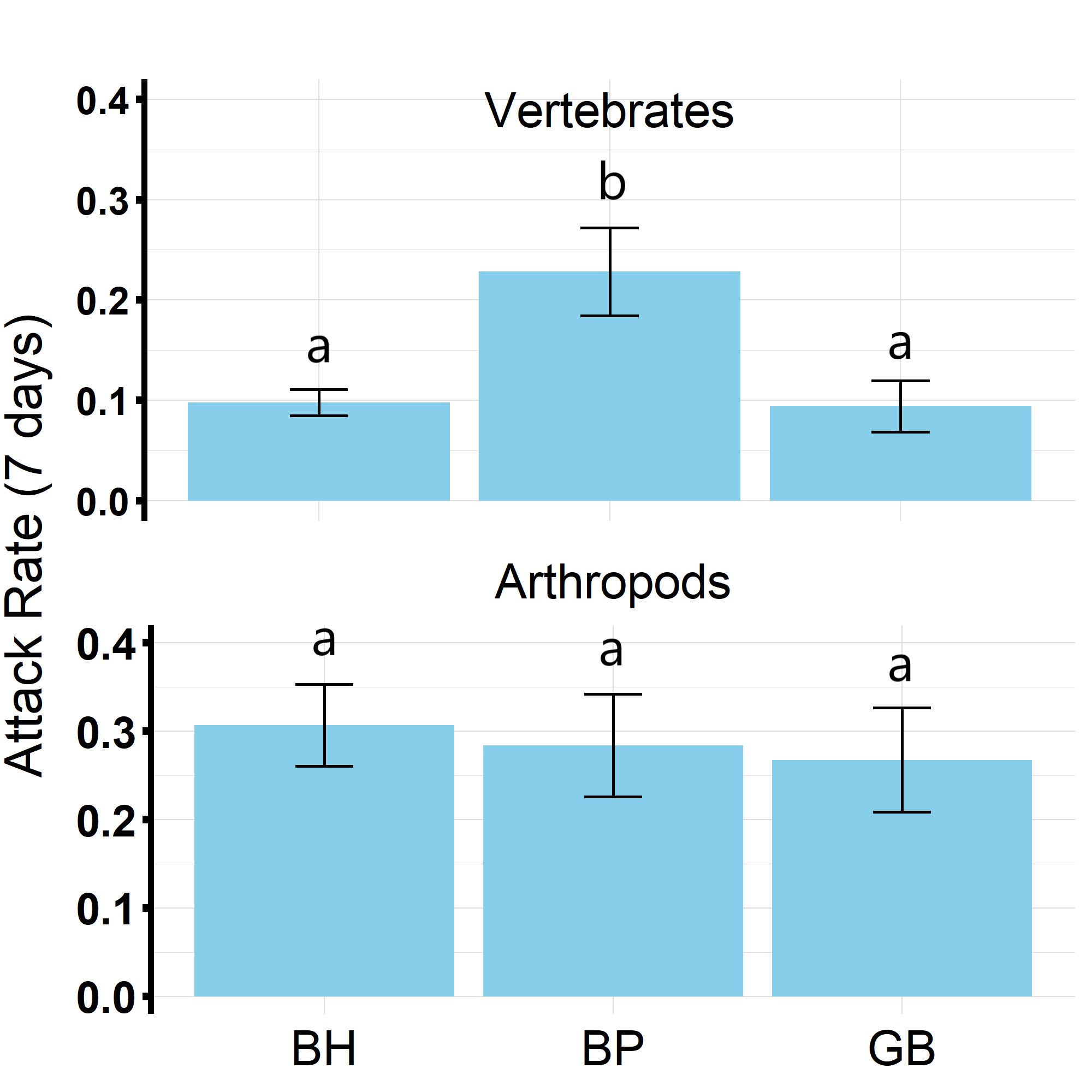


**Arthropod and vertebrate attack rates by site.** A) Attack rates (mean ± SE) by vertebrates (top panel) and arthropods (bottom panel) at each of the three sites averaged across all six trials. Vertebrate attack rates were significantly higher at BP compared to other sites, while there were no significant differences for arthropods across sites. Abbreviations: BH (Breakheart), BP (Bradley Palmer) and GB (Great Brook).

When looking at site variation, predation was about twice as high for vertebrates at BP than at the other two sites, whereas predation by arthropods did not vary among sites. Interestingly, BP was the site with greatest abundance and richness of insectivorous birds based on our bird point counts. Camera trap data also suggest that bird predator pressures at BP are greater than at GB [with more species: 10 vs 7.5 (5 x 1.5 = 7.5, # of species multiplied by 1.5 for the corrected number of cameras because GB had 2 cameras while BP had 3, and more individuals: 36 vs 10.5 (7 x 1.5 = 10.5)]. Our findings are supported by other studies that found that as bird abundance and richness increase, so do predation rates on insect herbivores (Bereczki *et al.,* 2014; Gonzalez-Gomez *et al.,*2006). The camera trap data for mammals is different. The data suggest that BP had about the same species as GB (8 vs 7.5 [5 x 1.5]) and a greater number of individuals (141 vs 213 [142 x 1.5]). However, the contribution of these mammals to the predation attempts depends on the degree to which the Eastern gray squirrels, Eastern chipmunks and *Peromyscus* are predators, which we were unable to ascertain from bite marks. These sites are all relatively similar to each other, but based on satellite imagery, BP may have less surrounding residential and commercial land than GB and BH. However, there is no clear ecological or anthropogenic factor that could account for greater bird diversity and predation at BP compared to the other sites.
